# Supplementary material for: Chimeric design of pyrrolysyl-tRNA synthetase/tRNA pairs and canonical synthetase/tRNA pairs for genetic code expansion
Source: Nat Commun. 2020 Jun 22;11:3154. doi: 10.1038/s41467-020-16898-y (PMC7308279; doi:10.1038/s41467-020-16898-y)
Supplement: Supplementary file 1 — Supplementary Information [file 41467_2020_16898_MOESM1_ESM.pdf]

Supplementary Information for:

Ding et al.

Chimeric design of Pyrrolysyl-tRNA synthetase/tRNA pairs and canonical synthetase/tRNA pairs for genetic code expansion

➢ **Supplementary Methods**

1 Chemical synthesis

2 Construction of vectors bearing chimeric tRNAs or chimeric synthetases

3 LC-MS/MS analysis of protein

4 Procedures for click reactions

5 Expression, purification, and fluorescence spectroscopy of HdeA with 6CNW or 7CNW incorporated

6 Expression and purification of ubiquitin variants

7 Chemical synthesis of 6CNW and 7CNW

➢ **Supplementary Table and Figures**

Supplementary Table 1. Purified protein yields of GFP variants

Supplementary Figure 1. The amino acids used in this study

Supplementary Figure 2. The cloverleaf structures of tRNAs used in this study

Supplementary Figure 3. The chimeric histidine system

Supplementary Figure 4. The expression of ubiquitin proteins with the chimeric systems

Supplementary Figure 5. Structural superposition of protein complex model using PyMOL

Supplementary Figure 6. Optimization of the chimeric histidyl-tRNA synthetase

Supplementary Figure 7. Nonsense suppression efficiency with the chimeric histidine system

Supplementary Figure 8. FACS analysis of amber suppression efficiency with the chimeric system

Supplementary Figure 9. Testing the activity of the chimeric histidine system in mammalian cells

Supplementary Figure 10. Testing the orthogonality of the chimeric histidine synthetase

Supplementary Figure 11. Engineering additional orthogonal aaRS/tRNA pairs with the chimera design

Supplementary Figure 12. The expression level of chimeric synthetases and their corresponding catalytic domains

Supplementary Figure 13. The chimeric phenylalanine system
Supplementary Figure 14. Testing the orthogonality of the chimeric phenylalanine and alanine synthetases
Supplementary Figure 15. The chimeric alanine system
Supplementary Figure 16. The chimeric serine system
Supplementary Figure 17. Incorporation of phenylalanine analogues with the chimeric phenylalanine system
Supplementary Figure 18. FACS analysis of amber suppression efficiency with the chimeric phenylalanine system
Supplementary Figure 19. Incorporation of L-Dopa with the chimeric phenylalanine system
Supplementary Figure 20. Incorporation of tryptophan analogues with the chimeric phenylalanine system
Supplementary Figure 21. The importance of E391D mutation in chPheRS-3
Supplementary Figure 22. Incorporation of 6CNW and 7CNW with the chimeric phenylalanine system
Supplementary Figure 23. Chemical synthesis of 6CNW and 7CNW
Supplementary Figure 24-46. NMR and HRMS data of synthetic compounds

Supplementary Methods

Supplementary Methods 1. Chemical synthesis

Unless otherwise noted, all solvents and other reagents were commercially available and used without further purification. Thin-layer chromatography (TLC) employed glass 0.25 mm silica gel plates. Flash chromatography columns were packed with 300-400 mesh silica gel in petroleum ether. Nuclear magnetic resonance spectra (^{1}H and ^{13}C NMR) were recorded on Varian Mercury-500 spectrometers. For ^{1}H measurements, the chemical shift refers to TMS or H_2O, showing a signal at 0 ppm and 3.33 ppm, respectively. As an internal standard, the remaining protons or carbons of the corresponding deuterated solvent were used (DMSO-d_6, 2.49 ppm (^{1}H-NMR), 39.52 ppm (^{13}C-NMR)). Multiplicities are denoted as follows: s = singlet, d = doublet, t = triplet, q = quartet, dd = doublet of doublets, and m = multiplet. Coupling constants (*J*) were reported in Hertz (Hz). High-resolution mass spectra (HRMS) were performed on Agilent 6200 TOF/ 6500 Q-TOF mass spectrometer (for cation) and AB Triple TOF 5600^{plus} mass spectrometer (for anion) by ESI.

Supplementary Methods 2. Construction of vectors bearing chimeric tRNAs or chimeric synthetases

In general, the chimeric tRNA and GFP-190TAG-His_6 genes were chemically synthesized and inserted into the pNEG vector by Gibson assembly. The chimeric tRNAs were driven by the *lpp* promoter. And the GFP gene was controlled by the araBAD promoter. The chimeric synthetases were either chemically synthesized or directly amplified from the genome, and then inserted into the pBK vector by Gibson assembly under the control of constitutive *glnS* promoter. The linkers in these chimeric synthetases were inserted into pBK vector by Q5 Site-Directed Mutagenesis Kit (New England BioLabs). For the generation of chimeric synthetases under the control of different promoters, araBAD promoter, *oxb20* promoter, and *trp* promoter were chemically

synthesized and inserted into pBK vectors by Gibson assembly to replace the *glnS* promoter.

Supplementary Methods 3. LC-MS/MS analysis of protein

For the LC-MS/MS analysis, the protein band was cut from the gel and sent to digestion with trypsin overnight. Trypsin digested peptides were analysed by LC-MS/MS on a Thermo Scientific Q Exactive Orbitrap mass spectrometer in conjunction with a Proxeon Easy-nLC II HPLC (Thermo Fisher Scientific) and Proxeon nanospray source. The digested peptides were loaded into a 100-micron x 20mm Magic C18 100Å 5U reverse phase trap where they were desalted online before being separated with a 75-micron x 100mm Magic C18 200Å 3U reverse phase column. Peptides were eluted using a 120 min gradient with a flow rate of 300 nL/min. An MS survey scan was obtained for the m/z range 300-1800, MS/MS spectra were acquired using a top 10 method, where the top 10 ions in the MS spectra were subjected to High Energy Collisional Dissociation (HCD). An isolation mass window of 2 m/z was for the precursor ion selection, and normalized collision energy of 30 eV was used for fragmentation. A 40 seconds duration was used for the dynamic exclusion.

The MS/MS raw files were processed with MaxQuant software[1] (version 1.6.14.0) with the embedded Andromeda search engine[2]. For the analysis of GFP-190-NAA, the searching was against the GFP protein sequence (UniProtKB - P42212) database, which consists of all 20 common amino acids at position 190 in GFP (protein sequence 166-214: KIRHNIEDGSVQLADHYQQNTPIG**X**GPVLLPDNHYLSTQSALSKDPNEK, X means 20 natural amino acids). In the program, cysteine carbamidomethylation was set as a fixed modification, and N-acetylation and methionine oxidation were set as variable modifications. The maximum modification was set to 5 per peptide. Trypsin/P specificity was required for the digestion mode, and the maximum allowed missed cleavages was set to 2. First search peptide tolerance was set to 20 ppm, and the main search peptide tolerance was 10 ppm. Peptides with a minimum of 7 amino acids and a maximum charge

of 7 were considered. The required FDR was set to 0.01 at peptide and protein levels. Other parameters followed the default settings. Peptides with MS/MS counts > 1 were considered to calculate the fidelity of desired natural amino acids incorporation with chimeric systems. For the analysis of GFP-39-UAA, L-dopa, 6-Cyano-Trp, and 7-Cyano-Trp were set as variable modification followed the same parameters above. The spectra of peptides were displayed by pLabel software (version 2.4)[3] with default parameters. All LC-MS/MS raw Data and the searching result are available via ProteomeXchange with identifier PXD018660.[4]

Supplementary Methods 4. Procedures for click reactions.

GFP containing site-specifically installed 4-Azido-Phe were labelled with the CuAAC reaction. The reactions were performed by the addition of 1 mM $CuSO_4$ (100x stock in water), 100 μM 2-(4-((bis((1-(tert-butyl)-1H-1,2,3-triazol-4-yl)methyl)amino)methyl)-1H-1,2,3-triazol1-yl)acetic acid (BTTAA, 100x stock in DMSO), 100 μM Cy3-alkyne (100x stock in DMSO, Click Chemistry Tools) and 2 mM sodium ascorbate (100x stock in water). The reactions were then agitated for 1 h at room temperature before quenching with 5 mM disodium bathocuproine disulfonate (BCS, 100x stock in water).

Supplementary Methods 5. Expression, purification, and fluorescence spectroscopy of HdeA with 6CNW or 7CNW incorporated.

To express HdeA proteins with 6CNW or 7CNW incorporated, the plasmid pNEG bearing the HdeA-38TAG and a chimeric tRNA and the plasmid pBK carrying the corresponding chimeric synthetase were co-transformed into chemically competent DH10B cells. The expression and purification were carried out as described above for GFP. After purification, the samples were dialyzed against NTA-0 buffer to remove imidazole. Fluorescence emission spectra were then measured on an FLSP920 Series of Fluorescence Spectrometers (Edinburgh Instruments).

Supplementary Methods 6. Expression and purification of ubiquitin variants

For ubiquitin expression and purification, the plasmid pNEG bearing the ubiquitin-S65TAG (pNEG bearing wild-type ubiquitin was made as a positive control) and a chimeric tRNA, and the plasmid pBK carrying the corresponding chimeric synthetase were co-transformed into chemically competent DH10B cells. Overnight cultured DH10B cells were diluted 1:100 into 100 ml of fresh LB medium that was supplemented with required antibiotics. The cells were grown until OD_{600} reaching ~0.8. L-arabinose was added with a final concentration of 0.2% to induce the expression of ubiquitin (30 °C, 220 rpm, 16 h). The cells were harvested by centrifuging at 3000 g for 20 min at 4 °C. The resulting cell pellets were suspended in ice-cold NTA-0 buffer (50 mM Tris-HCl, 150 mM NaCl,1mM DTT pH 7.4), and lyzed by sonication. The suspension was centrifuged at 10000 g for 60 min at 4 °C. The resulting supernatant was purified via Ni^{2+}-affinity chromatography on chelating Sepharose equilibrated with NTA-0 buffer and washed with NTA-0 buffer containing 30 mM imidazole. The proteins were eluted with NTA-0 buffer supplemented with 500 mM imidazole. Purified proteins were subjected to SDS-PAGE. Protein concentrations were determined by absorbance at 280 nm, and the extinction coefficients were predicted with the ProtParam tool (https://web.expasy.org/protparam/) for each protein.

Supplementary Table and Figures

Supplementary Table 1. Purified protein yields of GFP variants. GFP variants were expressed by the chimeric synthetase/tRNA pairs in *E. coli* DH10B cells at 30 °C. Purified protein yields were measured and listed in the Table. The wild-type GFP (190Asp) was used as a positive control for comparison.

GFP variants	Purified protein yields (mg/L)
GFP-wild type (190Asp)	28.6
GFP-190-His	37.5
GFP-190-Phe	13.6
GFP-190-Ala	14.5
GFP-190-Ser	4.0
GFP-190-4-Azido-Phe	8.1
GFP-190-2-Naphthyl-Ala	7.0
GFP-190-3-Cyano-Phe	2.3
GFP-190-L-Dopa	2.4
GFP-190-1-Methyl-Trp	1.3
GFP-190-6-Methyl-Trp	8.0
GFP-190-6-Chloro-Trp	4.5
GFP-190-6-Bromo-Trp	3.1
GFP-190-6-Cyano-Trp	1.6
GFP-190-7-Methyl-Trp	8.2
GFP-190-7-Chloro-Trp	5.1
GFP-190-7-Cyano-Trp	2.3

Supplementary Figure 1. The amino acids used in this study. The chemical structures and common names of amino acids used in this study are shown.

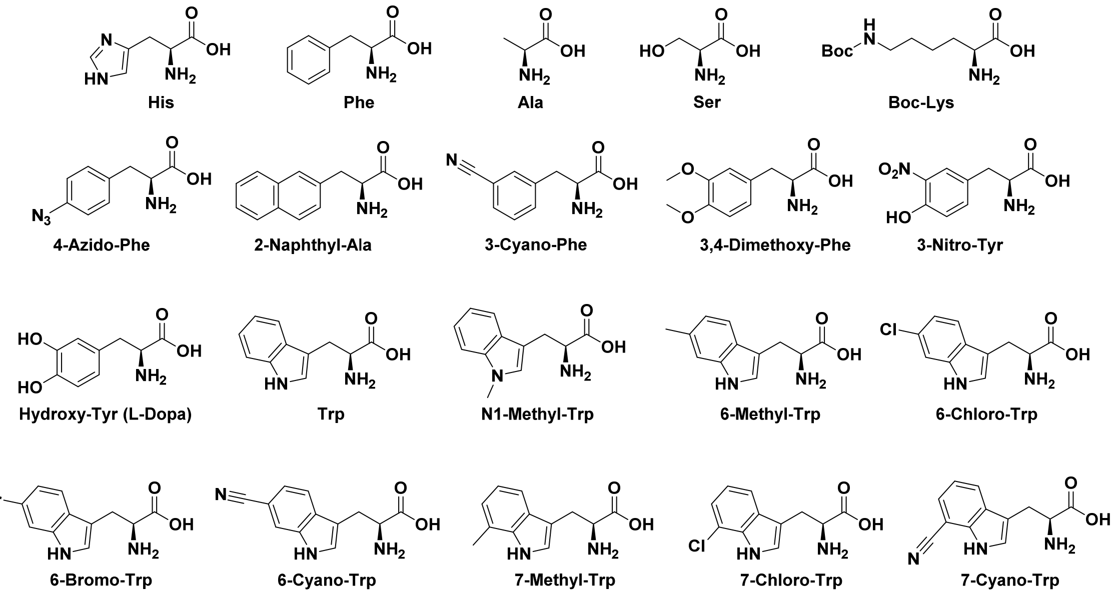

Supplementary Figure 2. The cloverleaf structures of tRNAs used in this study. (A) The structures of pylT and hisT used in this study. The pylT had a U25C mutation, which improved the efficiency of the pyrrolysine system. The hisT had a G^{-1} structure. (B) The structures of chimeric histidyl-tRNAs (chHisTs) from -1 to -6. In these chHisTs, the sequence from pylT is colored in green and the sequence from hisT is colored in blue and highlighted by dashed boxes. (C) The structures of histidyl-tRNA from *E. coli* and mammalian cells with a CUA anticodon. (D) The structures of other chimeric tRNAs used in this study, namely chPheT, chAlaTs. The chAlaT (G-U) had an unusual G-U pair colored in red, while the unusual pair was mutated to A-U pair in chAlaT (A-U). The chPheT had an unusual G-U pair in the acceptor arm, which was mutated to a G-C pair for the applications in mammalian cells. (E) The structures of chimeric seryl-tRNA -1 and -2. ChSerT-2 showed the highest amber suppression efficiency and was named as chSerT in the study.

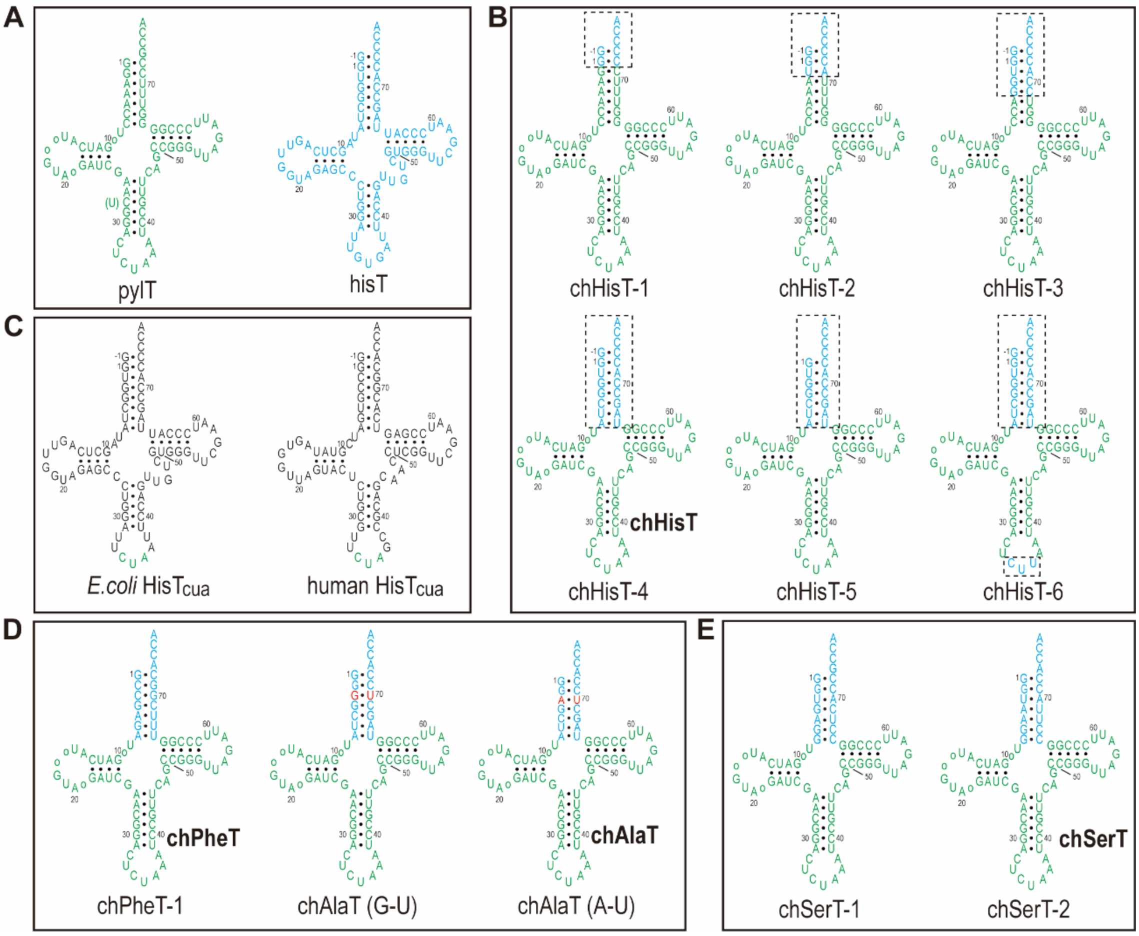

Supplementary Figure 3. The chimeric histidine system. (A) Coomassie blue staining gel analysis of purified GFP-190-His from indicated conditions. (B) Mass spectrometry characterization of the fidelity of His incorporation on GFP. The deconvoluted MS data was shown in the main text. (C) LC-MS/MS spectrometry characterization of the fidelity of His incorporation (>99%) on GFP. (D) The full native gel showed in Figure 3E, the region in the main text was highlighted by a red dashed-box. The wild-type GFP has a negative charge amino acid at the position 190 (Asp), which thus migrated faster than GFP variants in the native gel. (E) Western blotting analysis of amber suppression activity of the chimeric histidine system, when chHisRS is driven by promoter such as araBAD, *glnS*, *oxb20*, or *trp*. The truncated protein production is highlighted by a black dashed-box.

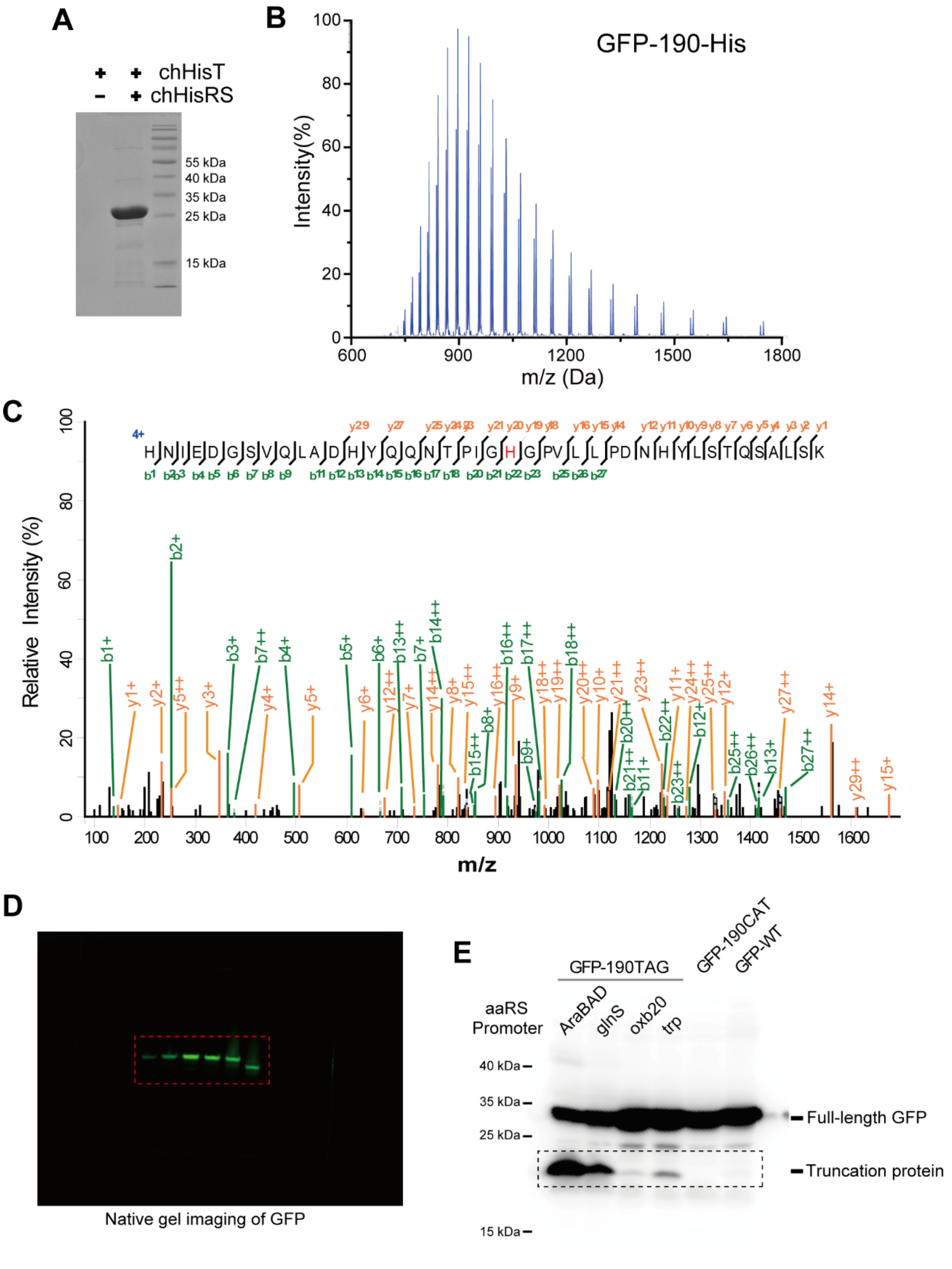

Supplementary Figure 4. The expression of ubiquitin proteins with the chimeric systems. (A) Coomassie blue staining gel analysis of purified full-length ubiquitin (Ub)

carrying Ser65TAG mutation from indicated chimeric synthetase/tRNA pairs with Ub-WT as a control. Purified protein yield is shown below the figure. (B) The full Coomassie blue staining gel showed in Supplementary Figure 4A, the region in Supplementary Figure 4A was highlighted by a red solid-box. The experiment in the figure was repeated twice with similar results.

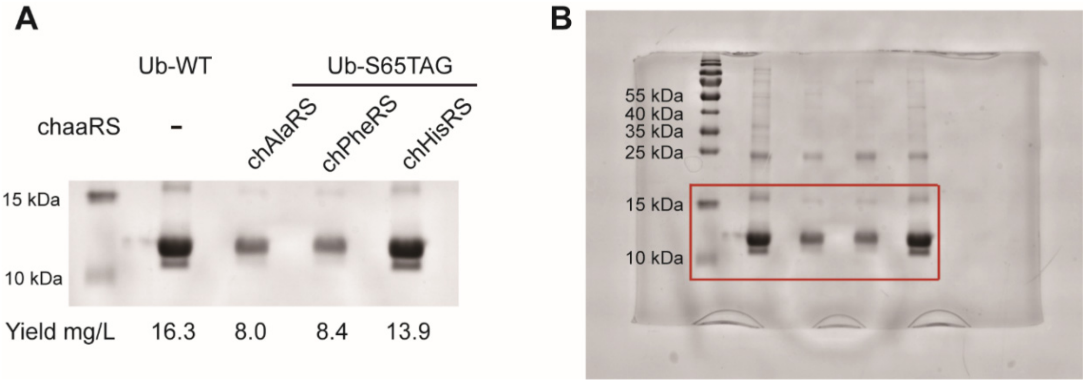

Supplementary Figure 5. Structural superposition of protein complex model using PyMOL. Structural superposition of protein complex model from the crystal structures: PDB, 5ud5 (protein colored in green, tRNA colored in magenta); PDB, 2zni (protein colored in limon, tRNA colored in cyan) and PDB, 4rdx (protein colored in blue, tRNA colored in orange), by overlaying the tRNA structures in these protein complex model using PyMOL.

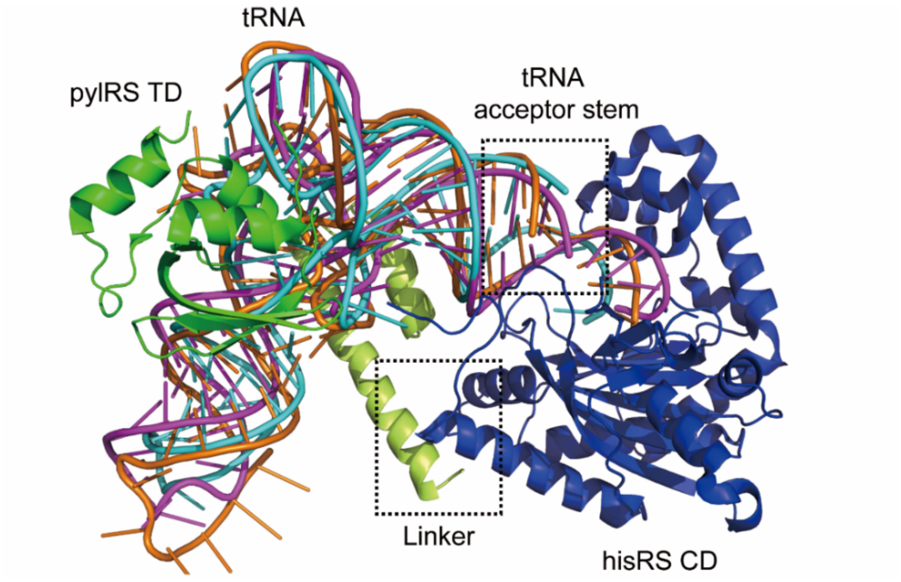

Supplementary Figure 6. Optimization of the chimeric histidyl-tRNA synthetase. (A) The cartoon structures of chHisRSs with different linker-lengths. In these chHisRSs, the sequence from pylRS is colored in green and the sequence from hisRS is colored in blue. (B) Analysis of amber suppression activity of the generated chHisRSs carrying different linker-lengths (GS-rich linker) by GFP reporter assay and native fluorescent in-gel imaging assay. ChHisRS 4-3 was named as chHisRS in the study. Error bars represented ±standard error of the mean (n=3). Statistical significance is quantified with ordinary one-way ANOVA (p value is showed in the figure). The full native gel is available in the Source Data file.

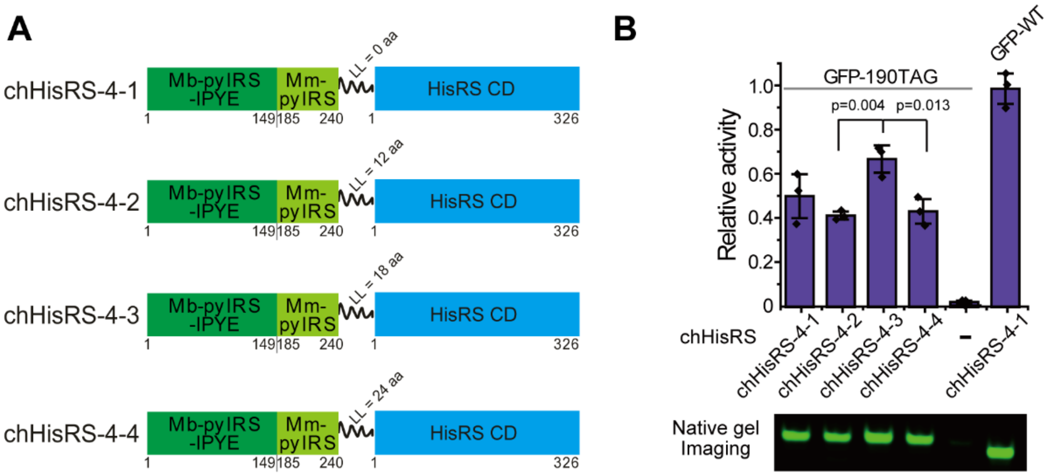

Supplementary Figure 7. Nonsense suppression efficiency with the chimeric histidine system. Analysis of the nonsense suppression activity of the GFP gene carrying one of the three stop codons (ochre, opal and amber codon) using the chimeric histidine system. The tRNA was mutated to generate corresponding anticodons for these stop codons. Error bars represented ±standard error of the mean (n=3).

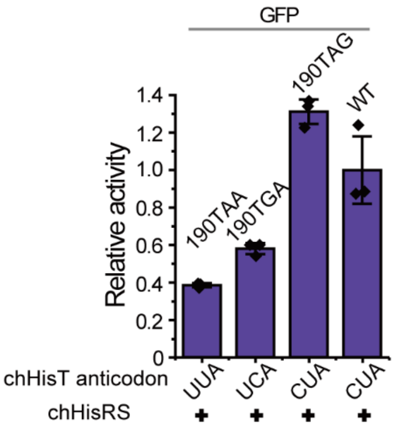

Supplementary Figure 8. FACS analysis of amber suppression efficiency with the chimeric systems. (A) Gating strategy to sort EGFP^{+}mCherry^{+} cells from HEK 293T cells in Fig. 5G-H and Supplementary Figure 8B, 18. The expressed cells expressed with fluorescent protein were used to set FITC and PE gains and gate. EGFP-WT cells and mCherry-WT cells as positive control to set FITC and PE gate were highlighted by black dashed-boxes (B) Flow cytometry analysis of amber suppression efficiency in mammalian cells transfected with the indicated chimeric systems. Full-length GFP expression was observed clearly in the presence of both the chimeric tRNA and the chimeric synthetase at a comparable level to the pyrrolysine system in incorporating BocK. In contrast, no detectable GFP expression was observed omitting the expression of the chimeric synthetases. For the BocK pylRS system, BocK (2 mM) is added to the medium of HEK 293T cells. For the chimeric systems, the addition of UAA is not necessary, and the corresponding amino acid in the medium is used as the substrate.

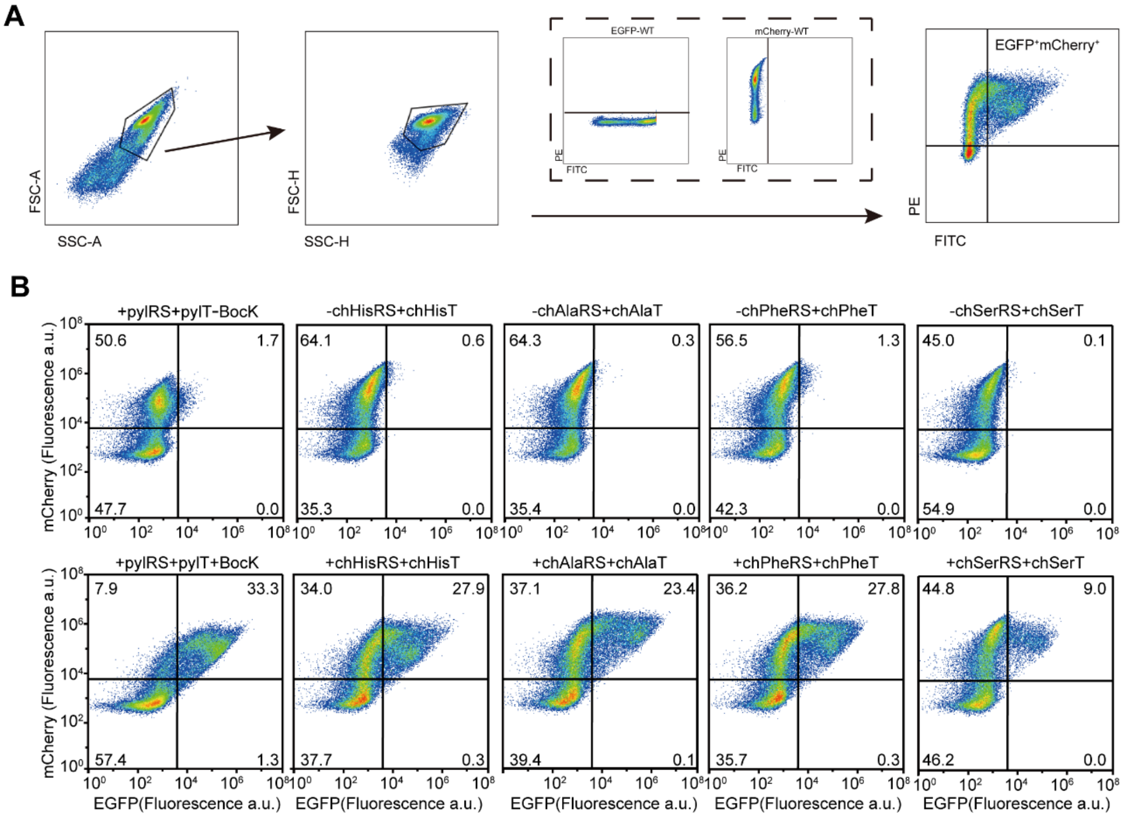

Supplementary Figure 9. **Testing the activity of the chimeric histidine system in mammalian cells.** (A) Western blotting analysis of the amber suppression efficiency of the chimeric histidine system in mammalian cells. (B), (C) The full western blotting membrane showed in Supplementary Figure 9A, the regions in Supplementary Figure 9A were highlighted by red dashed-boxes. The experiment in the figure was repeated twice with similar results.

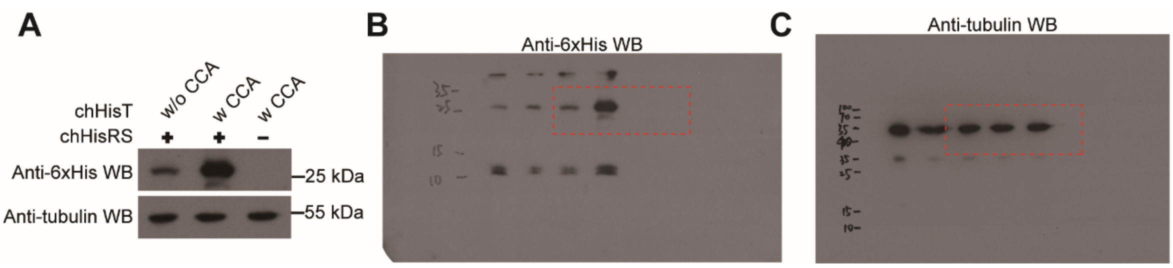

Supplementary Figure 10. Testing the orthogonality of the chimeric histidine synthetase. (A) For *in vitro* aminoacylation experiment, purified chHisRS is incubated with tRNAs extracted from *E. coli* cells (w/o the expression of chHisT) or mammalian cells. Purified HisRS or HisRS CD was used as control. (B) The relative amber suppression activity of endogenous HisTs carrying a CUA anticodon with the addition of chHisRS by GFP reporter assay. No additional GFP signal was detected in the presence of chHisRS using these endogenous HisTs. Error bars represented ±standard error of the mean (n=3 for the first two columns, n=4 for others).

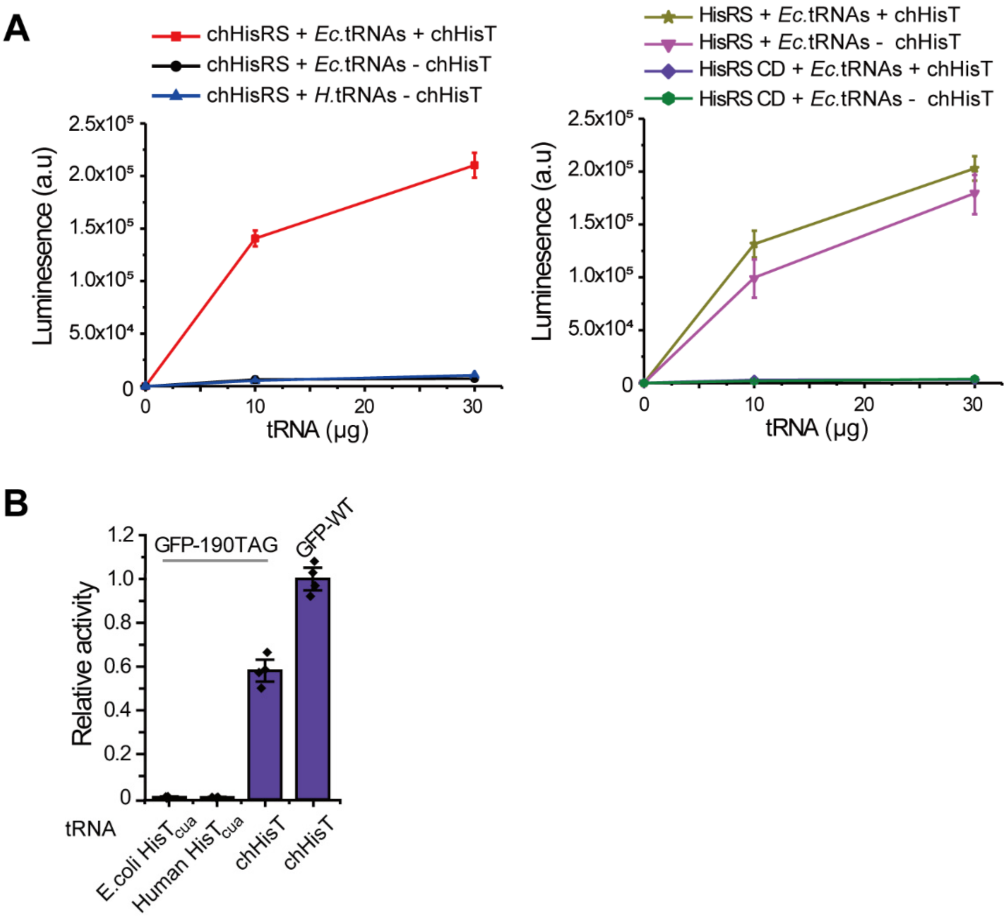

Supplementary Figure 11. Engineering additional orthogonal aaRS/tRNA pairs with the chimera design. (A) The cartoon structures of 16 additional chimeric aaRSs with sequence from pylRS colored in green and sequence from 16 common aaRSs in blue.

(B) Amber suppression activity of 16 additional chimeric aaRSs toward corresponding chT by GFP reporter assay, and the detailed information of 16 common aaRSs for construction of chimeric aaRSs. Ch*H.mt*.PheRS-1 in this figure was named as chPheRS in the study.

A

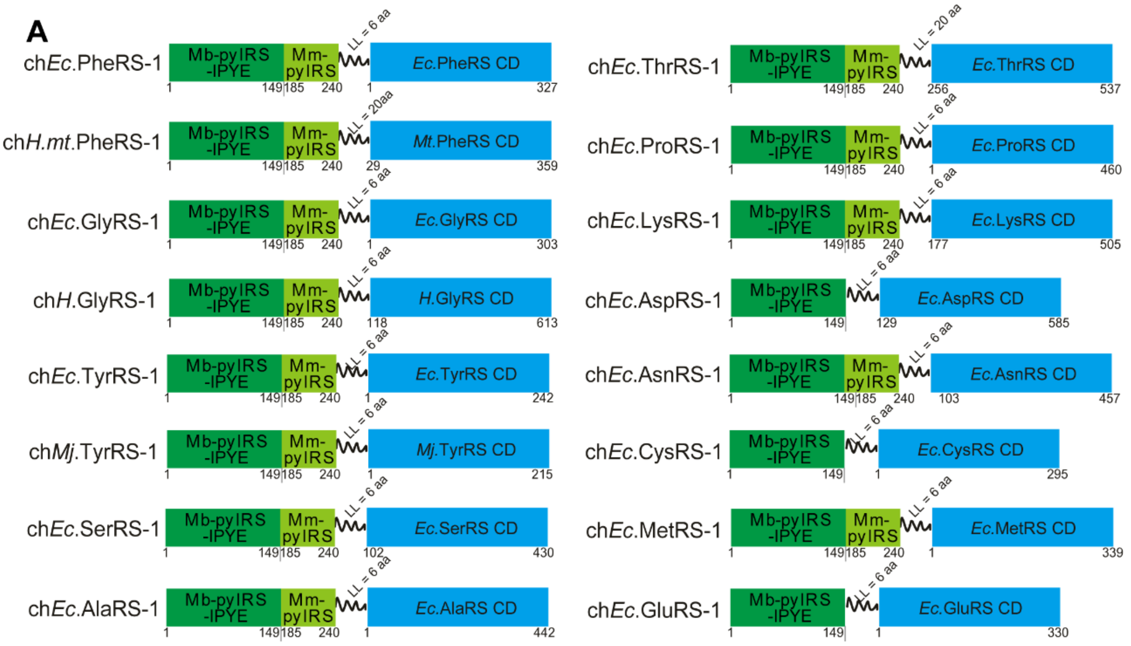

B

Name	Source	Type	Activity (%)	
			With chT	chT alone
ch*Ec*.PheRS-1	*E.coli*	IIc	0.04	0.01
ch*H.mt*.PheRS-1	*H.sapiens*	IIa	6.6	0.2
ch*Ec*.GlyRS-1	*E.coli*	IIc	8.3	12.5
ch*H*.GlyRS-1	*H.sapiens*	IIa	2.3	0.20
ch*Ec*.TyrRS-1	*E.coli*	Ic	1.2	0.47
ch*Mj*.TyrRS-1	*M.jannaschii*	Ic	0.56	0.36
ch*Ec*.SerRS-1	*E.coli*	IIa	1.6	0.41
ch*Ec*.AlaRS-1	*E.coli*	IIa	152	64.8
ch*Ec*.ThrRS-1	*E.coli*	IIa	0.33	0.63
ch*Ec*.ProRS-1	*E.coli*	IIa	0.43	0.32
ch*Ec*.LysRS-1	*E.coli*	IIb	0.33	0.93
ch*Ec*.AspRS-1	*E.coli*	IIb	2.0	0.26
ch*Ec*.AsnRS-1	*E.coli*	IIb	0.63	1.2
ch*Ec*.CysRS-1	*E.coli*	Ia	1.3	0.21
ch*Ec*.MetRS-1	*E.coli*	Ia	0.22	0
ch*Ec*.GluRS-1	*E.coli*	Ib	0.13	0

Supplementary Figure 12. The expression level of chimeric synthetases and their corresponding catalytic domains. The indicated chimeric synthetases and their corresponding catalytic domains were constructed with an N-terminus Flag tag under *glnS* promoter. The expression level of these proteins was detected by the anti-Flag western blotting with long exposure or short exposure time. The bands of the chimeric synthetases and the catalytic domains were highlighted by black dashed-boxes. The experiment in the figure was repeated twice and similar results were obtained.

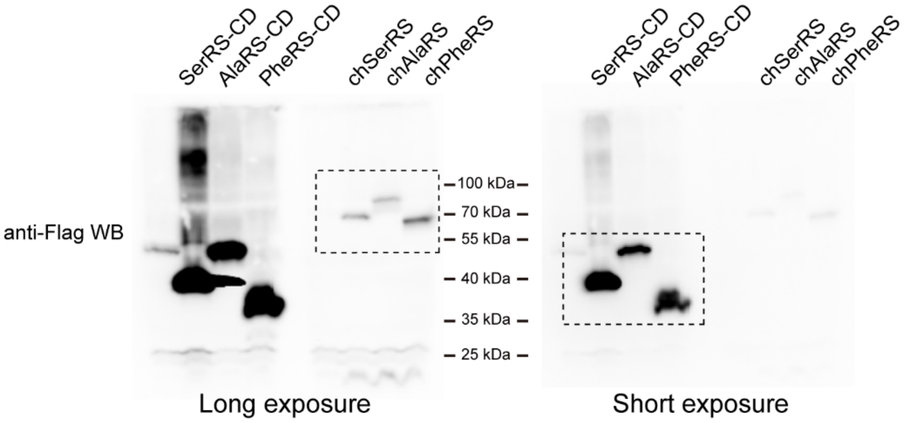

Supplementary Figure 13. The chimeric phenylalanine system. (A) Analysis of amber suppression activity of the generated chPheRSs by GFP reporter assay. (B) The full native gel showed in Figure 4B, the region in the main text was highlighted by a red dashed-box. (C) Coomassie blue staining gel analysis of purified GFP-190-Phe from indicated conditions. (D) Mass spectrometry characterization of the fidelity of Phe incorporation on GFP. The deconvoluted MS data was shown in the main text. (E) LC-MS/MS spectrometry characterization of the fidelity of Phe incorporation (>99%) on GFP. Ch*H.mt.*PheRS-1 in Supplementary Figure 11A was named as chPheRS in the study. In Figure 4B, chPheRS v1 and v2 were chPheRS that were driven by *glnS* and *oxb20* promoter, respectively. Error bars represented ±standard error of the mean (n=3).

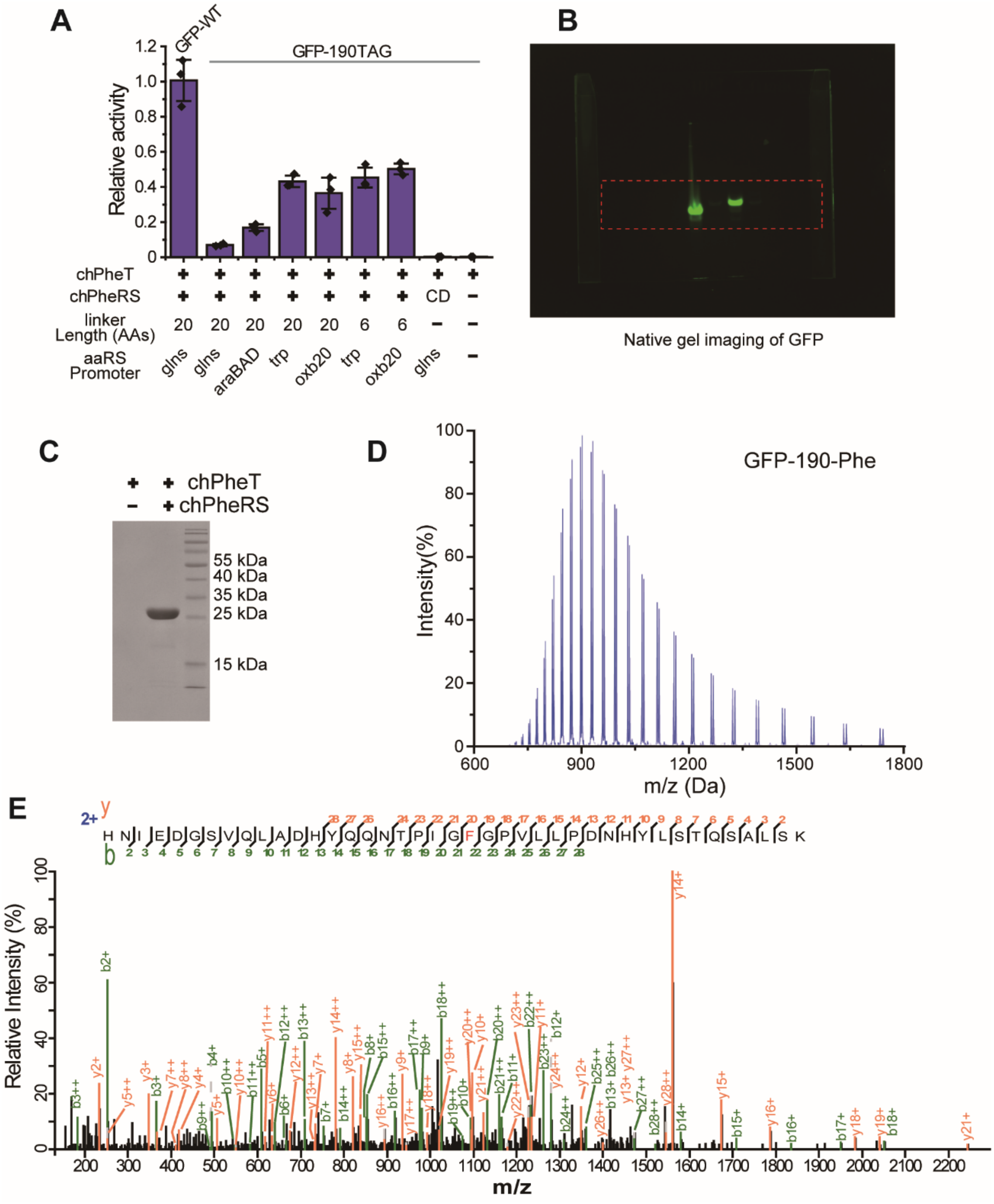
A
GFP-WT
GFP-190TAG
Relative activity
chPheT
chPheRS
linker Length (AAs)
aaRS Promoter
CD
glns
araBAD
trp
oxb20
B
Native gel imaging of GFP
C
chPheT
chPheRS
55 kDa
40 kDa
35 kDa
25 kDa
15 kDa
D
GFP-190-Phe
Intensity(%)
m/z (Da)
E
Relative Intensity (%)
m/z

Supplementary Figure 14. Testing the orthogonality of the chimeric phenylalanine and alanine synthetases. (A) Purified protein (chPheRS, PheRS or PheRS CD) was incubated with tRNAs extracted from *E. coli* cells (w/o the expression of chPheT) or mammalian cells with radiolabelled Phe. The aminoacylation reactions were determined by measuring the production of radioactive aminoacyl-tRNA (n=4). In the figure, PheRS was used as a positive control and PheRS CD was applied as a negative control. (B) Purified protein (chAlaRS, AlaRS or AlaRS CD) was incubated with tRNAs extracted from *E. coli* cells (w/o the expression of chAlaT) or mammalian cells with radiolabelled Ala. The aminoacylation reactions were determined by measuring the production of radioactive aminoacyl-tRNA (n=2). In the figure, AlaRS was used as a positive control and AlaRS CD was applied as a negative control. Error bars represented ±standard error of the mean.

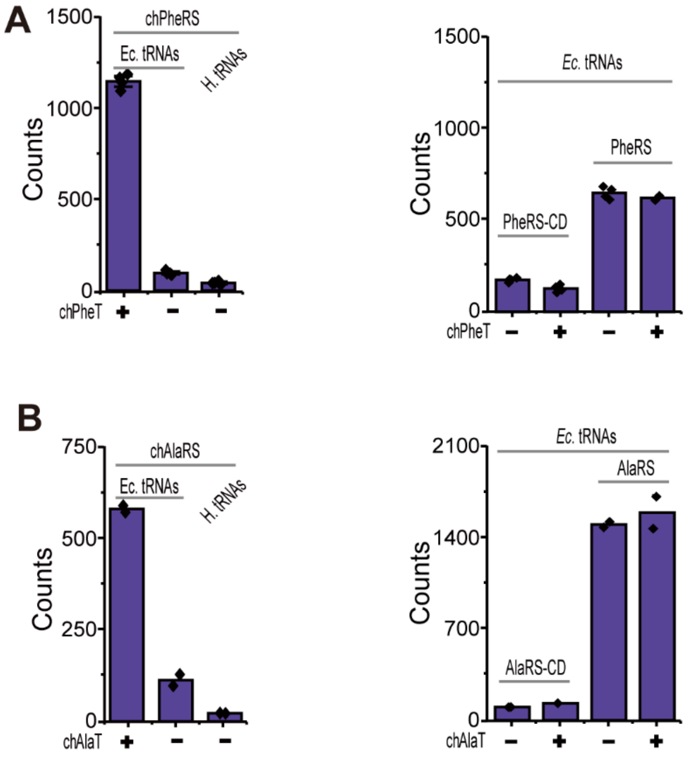

Supplementary Figure 15. The chimeric alanine system. (A) The cartoon structures of chimeric alanyl-tRNA synthetases (chAlaRSs) with the sequence from pylRS colored in green and the sequence from the alaRS colored in blue. The pylRS NTD mutant form carried IPYE mutations. (B) Analysis of amber suppression activity of the generated chAlaRSs towards chAlaT (G-U) by GFP reporter assay. (C) Analysis of amber suppression activity of the generated chAlaRSs towards chAlaT (A-U) by GFP reporter assay. ChAlaRS-4 was named as chAlaRS in the study. (D) The full native gel showed in Figure 4E, the region in the main text was highlighted by a red dashed-box. (E) Coomassie blue staining gel analysis of purified GFP-190-Ala from indicated conditions, chAlaT was chAlaT (A-U) in this figure. (F) Mass spectrometry characterization of the fidelity of Ala incorporation on GFP. The deconvoluted MS data was shown in the main text. (G) LC-MS/MS spectrometry characterization of the fidelity of Ala incorporation on GFP. Except for the incorporation of desired Ala (~97%), the incorporation of Gln (~1.2%) and Tyr (~1.6%) were also detected from MS/MS spectrometry. In Figure 4E, chAlaRS v1 and v2 were chAlaRS that were driven by *glnS* and *oxb20* promoter, respectively. Error bars represented ±standard error of the mean (n=3).

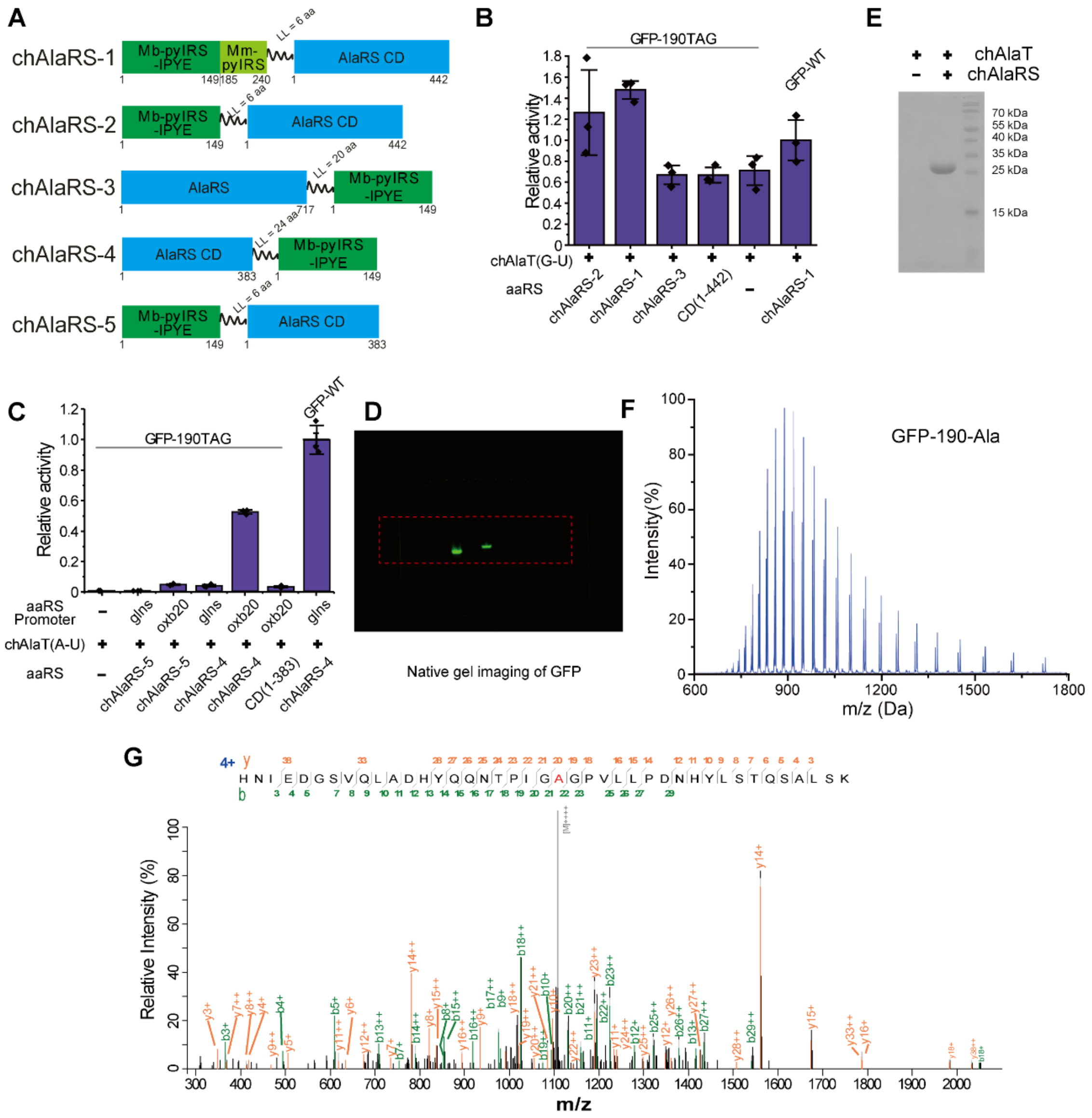
A
chAlaRS-1
chAlaRS-2
chAlaRS-3
chAlaRS-4
chAlaRS-5
Mb-pylRS -IPYE
Mm-pylRS
AlaRS CD
AlaRS
LL = 6 aa
LL = 20 aa
LL = 24 aa
B
GFP-190TAG
GFP-WT
Relative activity
chAlaT(G-U)
aaRS
chAlaRS-2
chAlaRS-1
chAlaRS-3
CD(1-442)
E
chAlaT
chAlaRS
70 kDa
55 kDa
40 kDa
35 kDa
25 kDa
15 kDa
C
GFP-190TAG
GFP-WT
Relative activity
aaRS Promoter
glns
oxb20
chAlaT(A-U)
aaRS
chAlaRS-5
chAlaRS-4
CD(1-383)
D
Native gel imaging of GFP
F
GFP-190-Ala
Intensity(%)
m/z (Da)
G
HNIEDGSVQLADHYQQNTPIGAGPVLLPDNHYLSTQSALSK
Relative Intensity (%)
m/z

Supplementary Figure 16. The chimeric serine system. (A) Mutation of chSerT-1 to chSerT-2. (B) Analysis of amber suppression activity of the generated chSerTs with chSerRSs by GFP reporter assay. Ch*Ec*.SerRS-1 in Supplementary Figure 11A was named as chSerRS in the study. In this figure, chSerRS v1 and v2 were chSerRS that were driven by *glnS* and *oxb20* promoter, respectively. (C) Mass spectrometry characterization of the fidelity of Ser incorporation on GFP. (D) LC-MS/MS spectrometry characterization of the fidelity of Ser incorporation (>99%) on GFP. Error bars represented ±standard error of the mean (n=3).

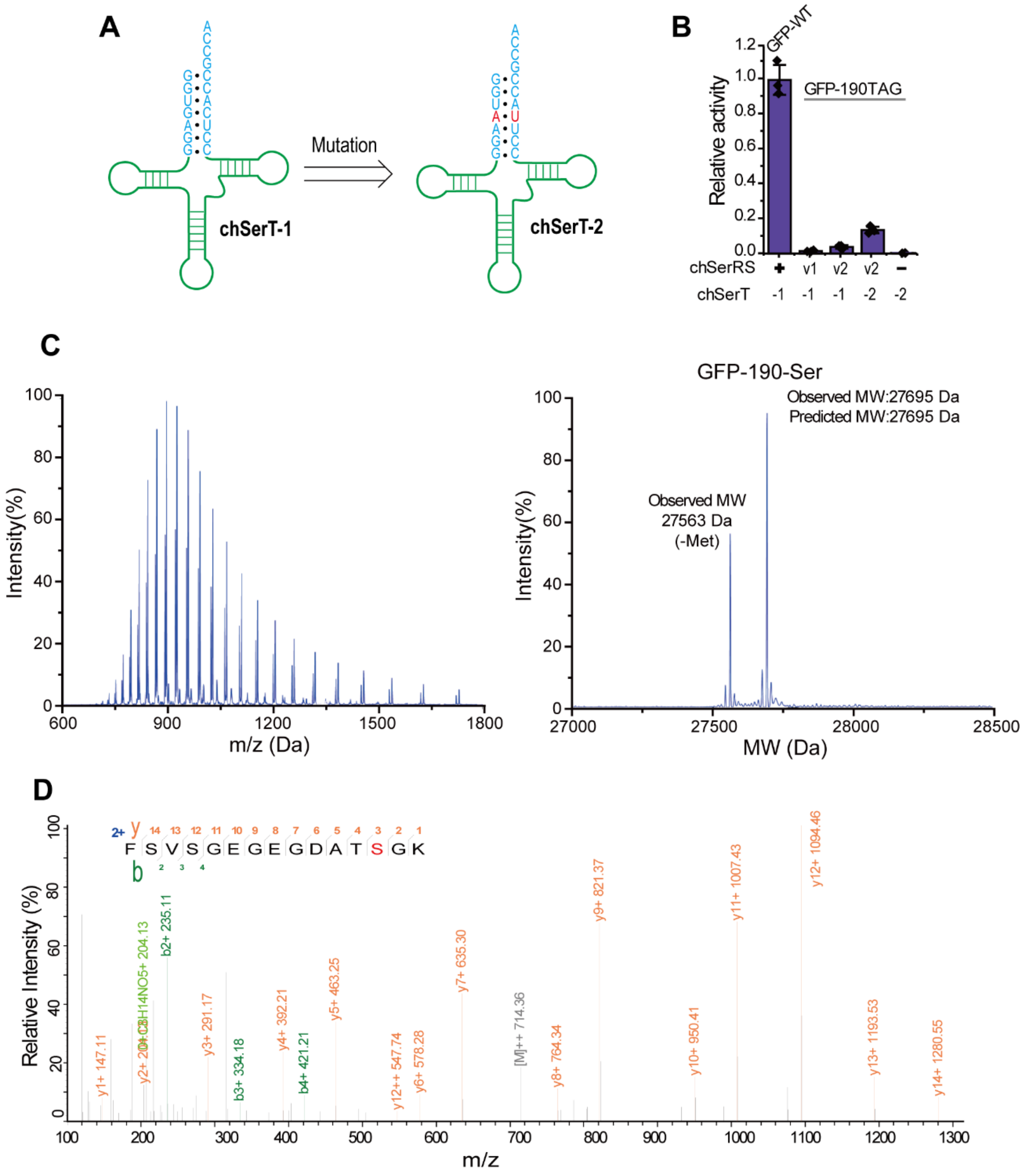

Supplementary Figure 17. Incorporation of phenylalanine analogues with the chimeric phenylalanine system. (A) GFP containing site-specifically installed 4-Azido-Phe by the indicated chPheRS variants were labelled with Cy3-alkyne with $Cu(BTTAA)_2$ as ligand and ascorbic acid (A.A) as reducing agent. Wild-type GFP was used as a negative control. The loading of GFP protein was shown on the Coomassie blue staining

gel. (B) The cell lysate of DH10B cells expressed with indicated components was labelled with Cy3-alkyne using Cu(BTTAA)$_2$ as ligand and A.A as reducing agent. Purified GFP-190-4-Azido-Phe was used as a positive control. Labelled samples were analyzed by the in-gel fluorescence imaging assay with long exposure or short exposure time. Coomassie blue staining was performed as the protein loading control. (C-E) Mass spectrometry characterization of the fidelity of Phe analogues incorporation into GFP by chPheRS-1.

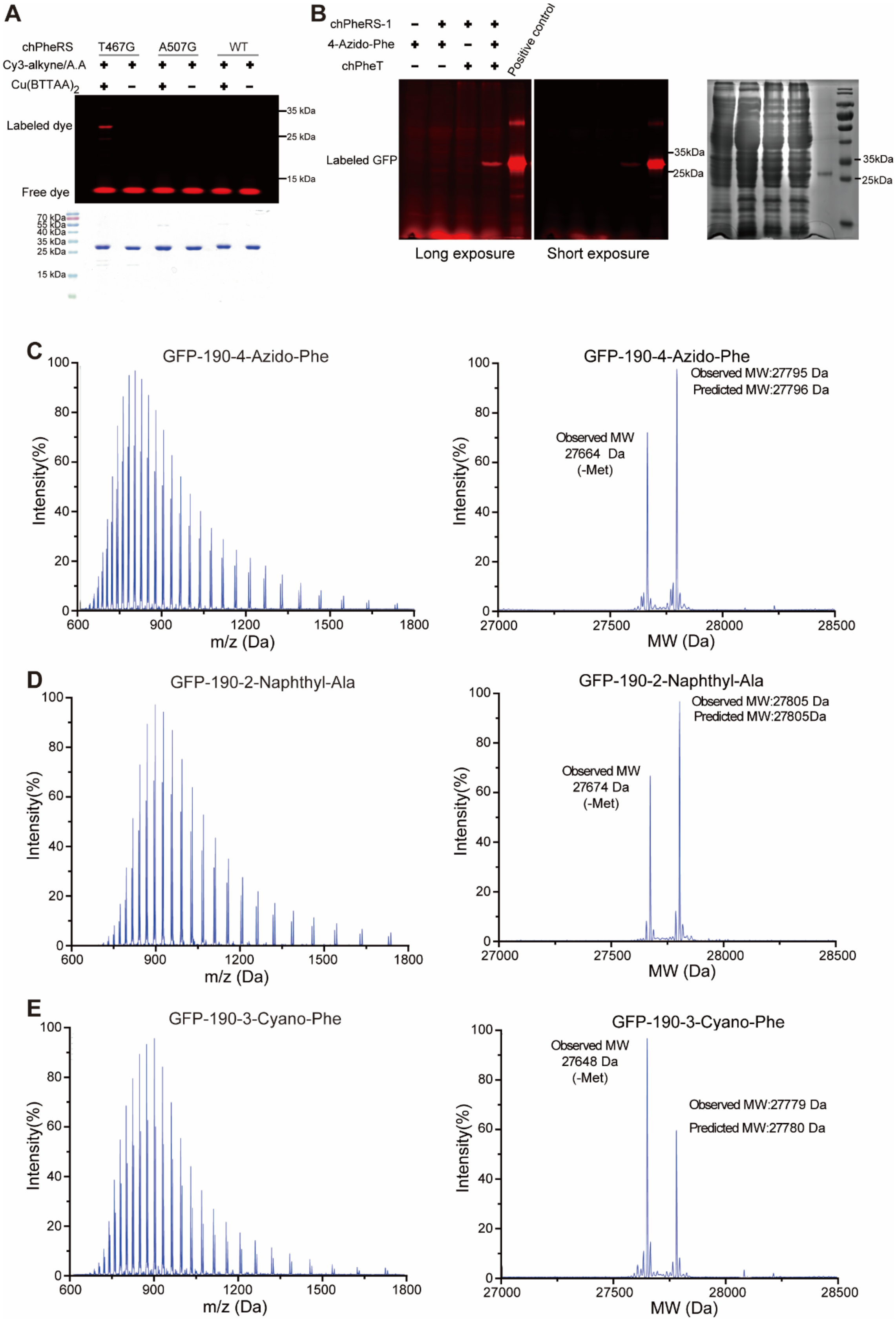
A
chPheRS
T467G
A507G
WT
Cy3-alkyne/A.A
Cu(BTTAA)2
Labeled dye
Free dye
35 kDa
25 kDa
15 kDa
70 kDa
55 kDa
40 kDa
B
chPheRS-1
4-Azido-Phe
chPheT
Positive control
Labeled GFP
35kDa
25kDa
Long exposure
Short exposure
C
GFP-190-4-Azido-Phe
Intensity(%)
m/z (Da)
Observed MW:27795 Da
Predicted MW:27796 Da
Observed MW
27664 Da
(-Met)
MW (Da)
D
GFP-190-2-Naphthyl-Ala
Observed MW:27805 Da
Predicted MW:27805Da
Observed MW
27674 Da
(-Met)
E
GFP-190-3-Cyano-Phe
Observed MW
27648 Da
(-Met)
Observed MW:27779 Da
Predicted MW:27780 Da

Supplementary Figure 18. FACS analysis of amber suppression efficiency with the chimeric phenylalanine system. Flow cytometry analysis of amber suppression efficiency of chPheRS-1 and chPheRS-3 in mammalian cells with or without the addition of 2 mM of indicated UAAs.

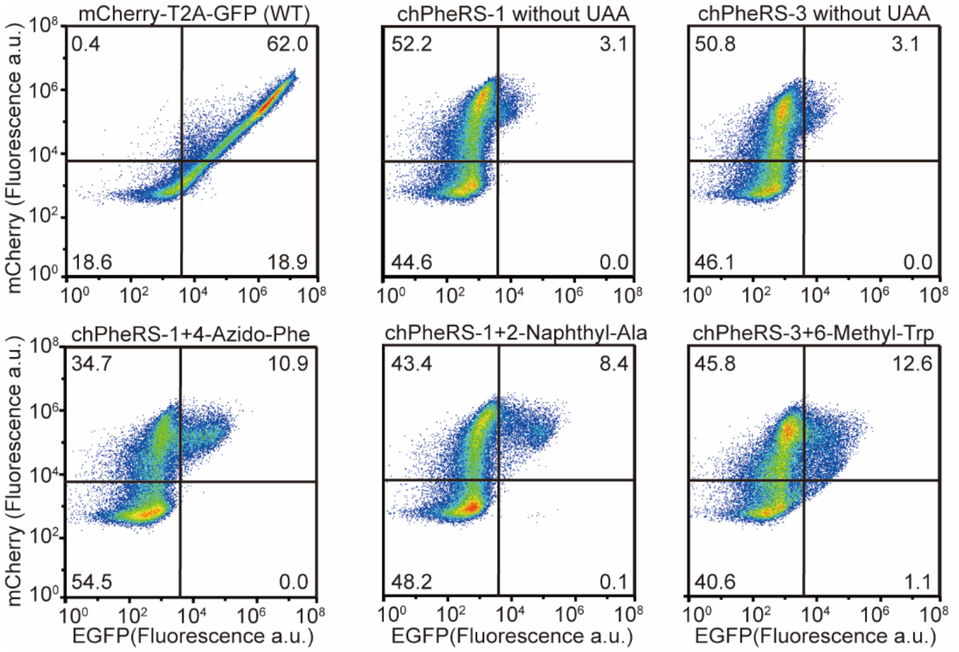

Supplementary Figure 19. Incorporation of L-Dopa with the chimeric phenylalanine system. (A) Structure of PheRS amino acid binding pocket with L-Dopa (PDB: 3ETG). The amino acids surrounding the hydroxyl groups of L-Dopa were highlighted in red. (B) Analysis of the amber suppression activity of the generated L-Dopa synthetase (chPheRS-2) with GFP reporter assay. Error bars represented ±standard error of the mean (n=3). (C) Mass spectrometry characterization of the fidelity of L-Dopa incorporation on GFP. (D) LC-MS/MS spectrometry characterization of L-Dopa incorporation on GFP. Because L-Dopa was oxidized easily to L-Dopaquinone during sample preparation for MS, the oxidized one was detected on the peptide by LC-MS/MS. (E) The amber suppression efficiency of L-Dopa synthetase (chPheRS-2) in mammalian cells with or without the addition of 1 mM L-Dopa. Scale bar: 50 μm.

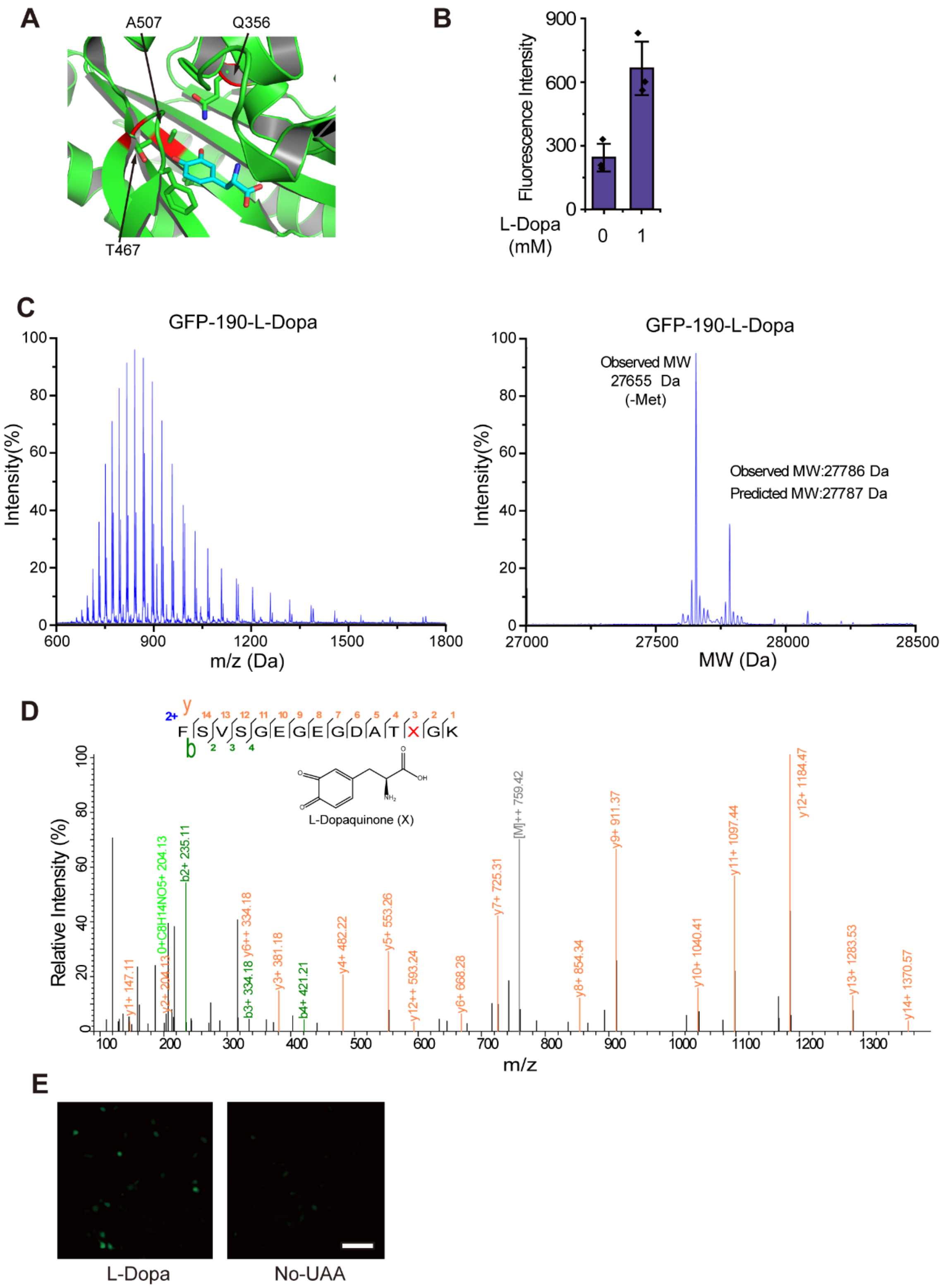
A
A507
Q356
T467
B
Fluorescence Intensity
L-Dopa (mM)
C
GFP-190-L-Dopa
Intensity(%)
m/z (Da)
Observed MW 27655 Da (-Met)
Observed MW:27786 Da
Predicted MW:27787 Da
MW (Da)
D
F S V S G E G E G D A T X G K
L-Dopaquinone (X)
Relative Intensity (%)
m/z
[M]++ 759.42
y12+ 1184.47
y9+ 911.37
y11+ 1097.44
b2+ 235.11
y7+ 725.31
y6++ 334.18
y5+ 553.26
y4+ 482.22
y3+ 381.18
y1+ 147.11
b3+ 334.18
b4+ 421.21
y10+ 1040.41
y13+ 1283.53
y14+ 1370.57
y8+ 854.34
y12++ 593.24
y6+ 668.28
E
L-Dopa
No-UAA

Supplementary Figure 20. Incorporation of tryptophan analogues with the chimeric phenylalanine system. (A-F) Mass spectrometry characterization of Trp analogues incorporation into GFP by chPheRS-3 or chPheRS-4. No additional UAA was added in Supplementary Figure 20G.

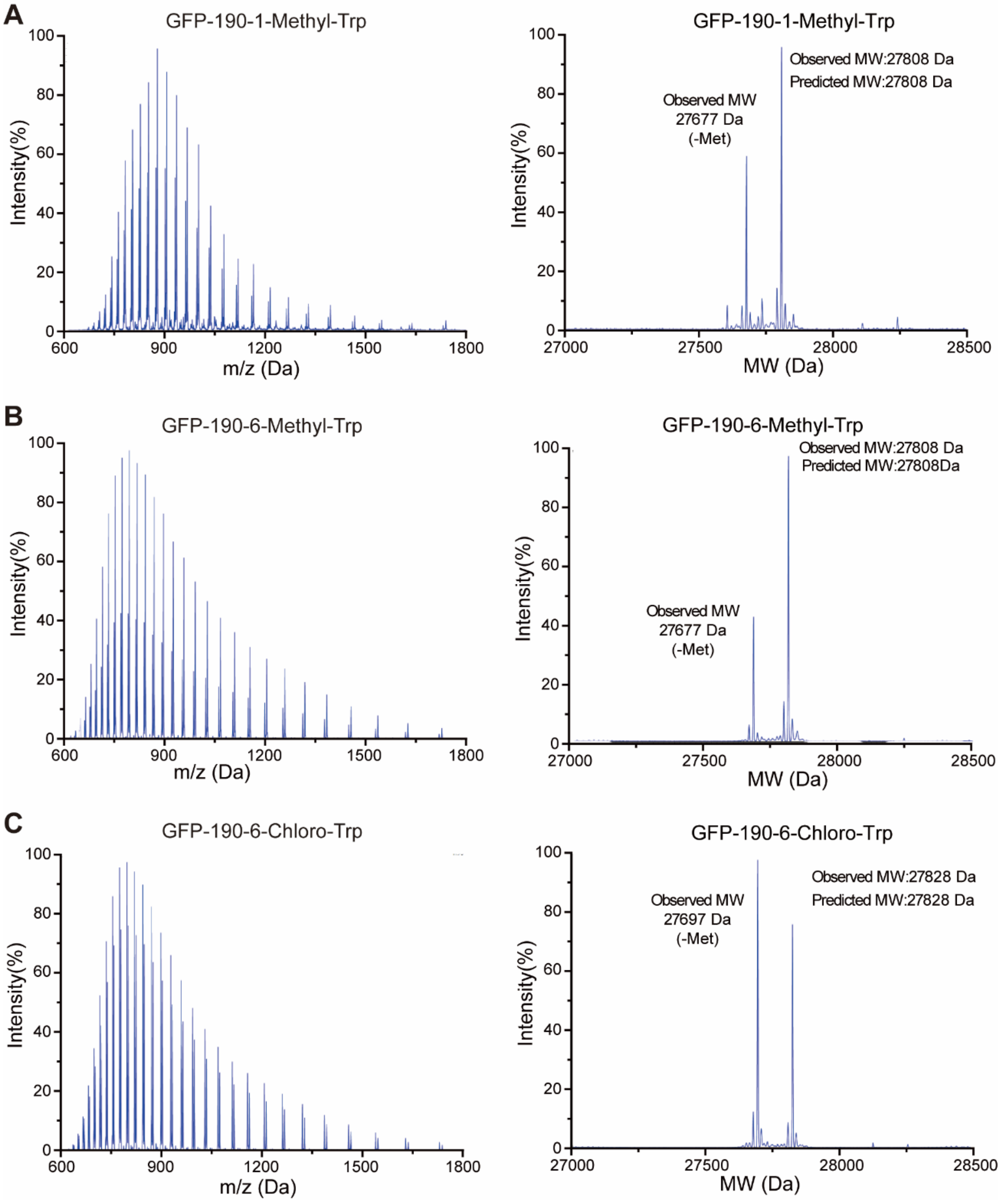

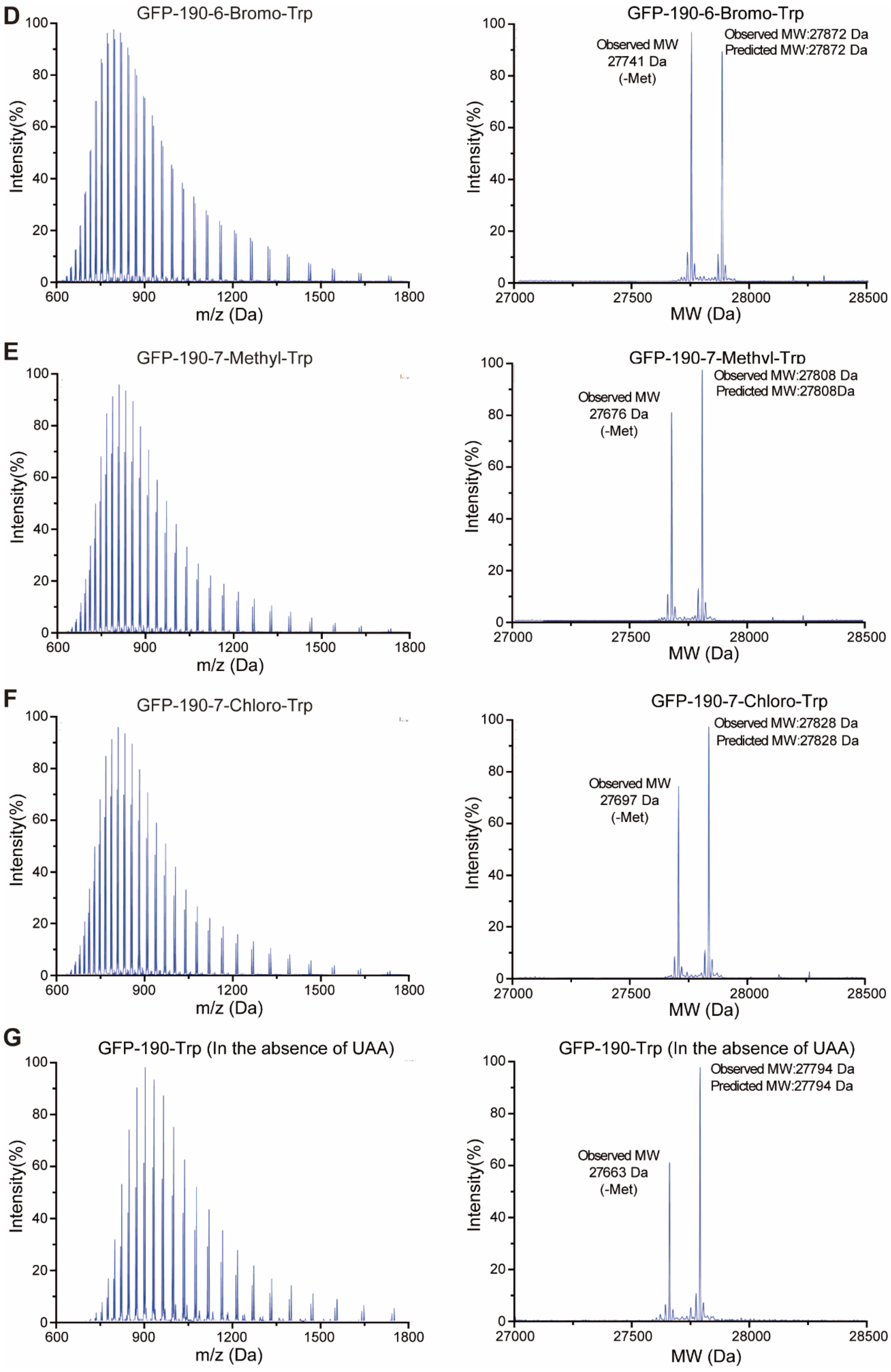
D
GFP-190-6-Bromo-Trp
Intensity(%)
m/z (Da)
GFP-190-6-Bromo-Trp
Observed MW
27741 Da
(-Met)
Observed MW:27872 Da
Predicted MW:27872 Da
Intensity(%)
MW (Da)
E
GFP-190-7-Methyl-Trp
Intensity(%)
m/z (Da)
GFP-190-7-Methyl-Trp
Observed MW:27808 Da
Predicted MW:27808Da
Observed MW
27676 Da
(-Met)
Intensity(%)
MW (Da)
F
GFP-190-7-Chloro-Trp
Intensity(%)
m/z (Da)
GFP-190-7-Chloro-Trp
Observed MW:27828 Da
Predicted MW:27828 Da
Observed MW
27697 Da
(-Met)
Intensity(%)
MW (Da)
G
GFP-190-Trp (In the absence of UAA)
Intensity(%)
m/z (Da)
GFP-190-Trp (In the absence of UAA)
Observed MW:27794 Da
Predicted MW:27794 Da
Observed MW
27663 Da
(-Met)
Intensity(%)
MW (Da)

Supplementary Figure 21. The importance of E391D mutation in chPheRS-3. Analysis of the amber suppression activity of the generated chPheRSs with the indicated mutation on E391 towards chPheT with GFP reporter assay. Error bars represented ±standard error of the mean (n=3).

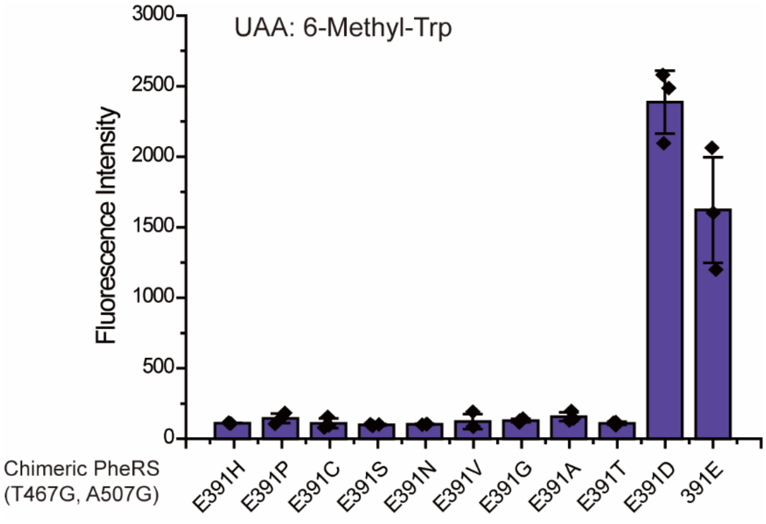

Supplementary Figure 22. Incorporation of 6CNW and 7CNW with the chimeric phenylalanine system. (A, B) Mass spectrometry characterization of 6CNW or 7CNW incorporation into GFP. The deconvoluted MS data was shown in the main text. (C, D) LC-MS/MS spectrometry characterization of 6CNW and 7CNW incorporation into GFP. (E) The fluorescence emission spectrum of HdeA proteins site-specifically incorporated with 6CNW or 7CNW at position 38. The proteins were excited with a 340 nm wavelength of light.

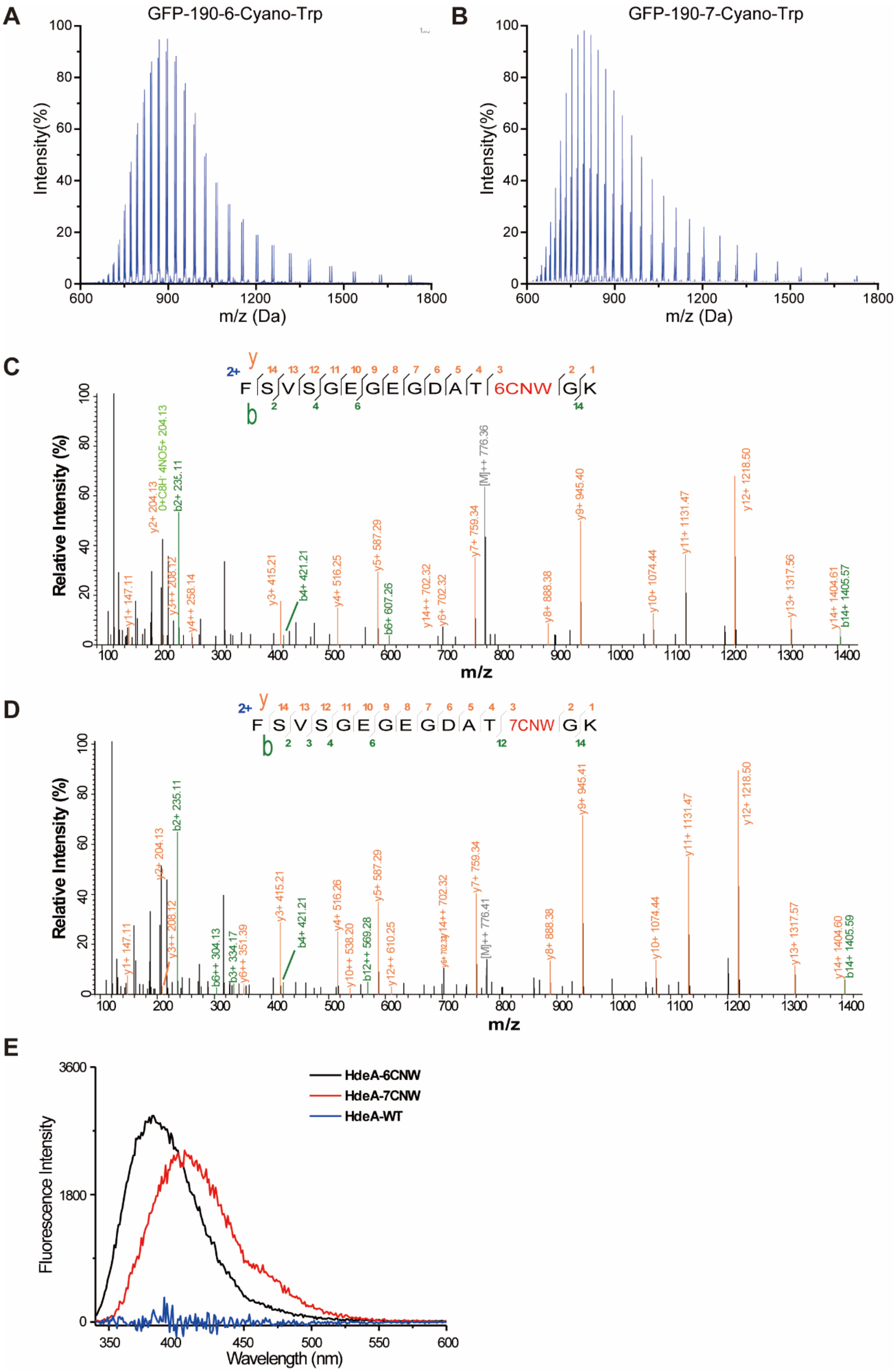
A
GFP-190-6-Cyano-Trp
Intensity(%)
m/z (Da)
B
GFP-190-7-Cyano-Trp
Intensity(%)
m/z (Da)
C
F S V S G E G E G D A T 6CNW G K
Relative Intensity (%)
m/z
D
F S V S G E G E G D A T 7CNW G K
Relative Intensity (%)
m/z
E
HdeA-6CNW
HdeA-7CNW
HdeA-WT
Fluorescence Intensity
Wavelength (nm)

Supplementary Methods 7. Chemical synthesis of 6CNW and 7CNW

Supplementary Figure 23. Chemical synthesis of 6CNW and 7CNW. A four-step synthetic route for Cyano-Trp is shown in the figure. The isolation yield of each synthetic step is highlighted under the product structure.

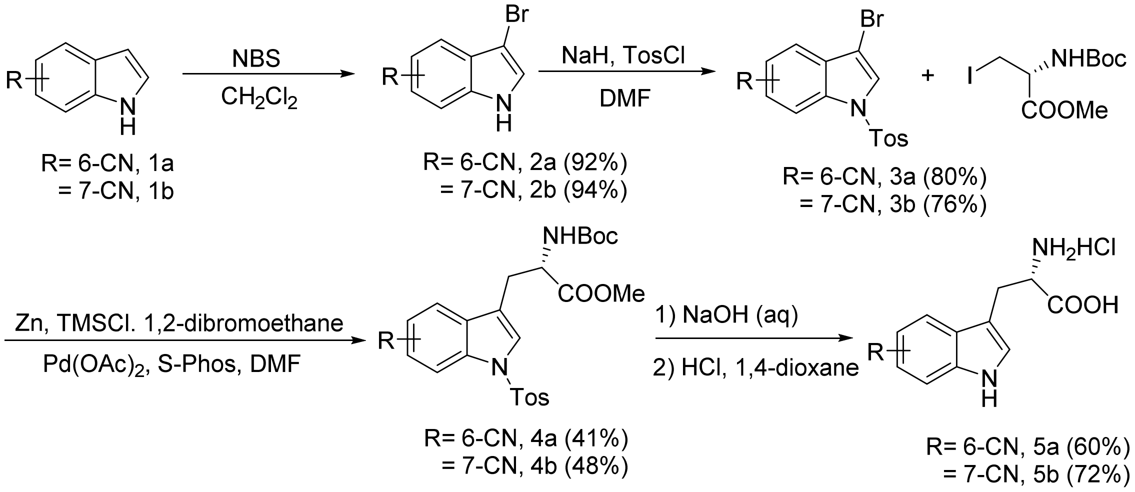

Preparation of Various Substituted 3-bromo-1*H*-indole (2)

3-bromo-1*H*-indole-6-carbonitrile (**2a**)   3-bromo-1*H*-indole-7-carbonitrile (**2b**)

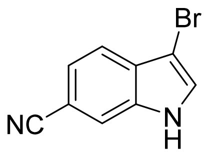

2a

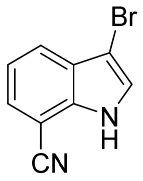

2b

To a solution of 1*H*-indole-6-carbonitrile or 1*H*-indole-7-carbonitrile (1.6 g, 11.25 mmol) in DCM (60 ml) was added NBS (2.0 g, 11.25 mmol) in portions over a period of 5 minutes at 25 °C. The resultant mixture was stirred at 25 °C for 1h. The reaction mixture was quenched with saturated sodium thiosulfate solution (25 ml) and further diluted with DCM (60 ml). The organic layer was washed water (2×25 ml) followed by brine solution and dried over anhydrous sodium sulfate, filtered and concentrated. The residue was purified by column chromatography using PE and EtOAc to afford the compound **2a** and

2b. **2a**) White solid (2.03 g, 92%). ^{1}H NMR (500 MHz, $CDCl_3$) δ 8.67 (s, 1H), 7.75 (s, 1H), 7.66 (d, *J* = 8.3 Hz, 1H), 7.45 (dd, *J* = 3.0, 2.0 Hz, 1H), 7.43 (d, *J* = 1.3 Hz, 1H) (**Supplementary Figure 24**). HRMS (ESI) m/z calcd. for $C_9H_4BrN_2^-(M\text{-}H)^-$ 218.9563, found 218.9573 (**Supplementary Figure 25**). **2b**) White solid (2.073 g, 94%). ^{1}H NMR (500 MHz, DMSO-d_6) δ 12.46 (s, 1H), 7.79 (d, *J* = 8.0 Hz, 1H), 7.76 (d, *J* = 2.6 Hz, 1H), 7.73 (dd, *J* = 7.4, 0.8 Hz, 1H), 7.29 (dd, *J* = 7.9, 7.5 Hz, 1H) (**Supplementary Figure 26**). ^{13}C NMR (126 MHz, DMSO-d_6) δ 135.27 (s), 128.29 (s), 127.63 (s), 124.35 (s), 120.62 (s), 117.27 (s), 95.07 (s), 90.54 (s) (**Supplementary Figure 27**). HRMS (ESI) m/z calcd. for $C_9H_4BrN_2^-(M\text{-}H)^-$ 218.9563, found 218.9571 (**Supplementary Figure 28**).

Preparation of Various Substituted 3-bromo-1-tosyl-1*H*-indole (3)

3-bromo-1-tosyl-1*H*-indole-6-carbonitrile (**3a**)

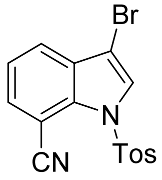

3a

3-bromo-1-tosyl-1*H*-indole-7-carbonitrile (**3b**)

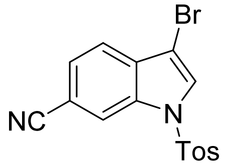

3b

NaH in oil (400 mg, 10 mmol) was washed with hexane and suspended in dry DMF (10 ml) under an Argon atmosphere. To this suspension was added the compound **2** (1.99 g, 9 mmol) in dry DMF (10 ml) at 0 °C. After the mixture was stirred for 10 min, tosyl chloride (1.9 g, 10 mmol) was added and the reaction mixture was stirred at 25 °C for 2

h. The mixture was poured into H_2O and extracted with EtOAc. The EtOAc layer was washed with brine, dried over Na_2SO_4, and concentrated. The residue was purified by column chromatography using PE and EtOAc to afford the compound **3a** and **3b**. **3a**) White solid (2.7 g, 80%). ^{1}H NMR (500 MHz, DMSO-d_6) δ 8.50 (s, 1H), 8.47 (s, 1H), 8.09 (d, J = 8.4 Hz, 2H), 7.78 (dd, J = 8.2, 1.3 Hz, 1H), 7.67 (d, J = 8.2 Hz, 1H), 7.45 (d, J = 8.3 Hz, 2H), 2.35 (s, 3H) (**Supplementary Figure 29**). ^{13}C NMR (126 MHz, DMSO-d_6) δ 146.97 (s), 133.78 (s), 132.99 (s), 132.69 (s), 131.06 (s), 129.93 (s), 127.92 (s), 127.77 (s), 121.54 (s), 119.35 (s), 118.13 (s), 108.62 (s), 98.83 (s), 21.57 (s) (**Supplementary Figure 30**). HRMS (ESI) m/z calcd. for $C_{16}H_{11}BrN_2NaO_2S^+$ $(M+Na)^+$ 396.9617, found 396.9614 (**Supplementary Figure 31)**. **3b**) White solid (2.565 g, 76%).^{1}H NMR (500 MHz, DMSO-d_6) δ 8.42 (s, 1H), 7.98 (d, J = 8.5 Hz, 2H), 7.95 (dd, J = 7.6, 1.0 Hz, 1H), 7.91 (dd, J = 8.0, 1.2 Hz, 1H), 7.58 – 7.53 (m, 1H), 7.49 (d, J = 8.6 Hz, 2H), 2.37 (s, 3H) (**Supplementary Figure 32**). ^{13}C NMR (126 MHz, DMSO-d_6) δ 146.80 (s), 134.34 (s), 134.24 (s), 131.72 (s), 130.99 (s), 130.86 (s), 129.43 (s), 128.01 (s), 126.22 (s), 125.13 (s), 116.89 (s), 98.60 (s), 97.94 (s), 21.60 (s) (**Supplementary Figure 33**). HRMS (ESI) m/z calcd. for $C_{16}H_{11}BrN_2NaO_2S^+$ $(M+Na)^+$ 396.9617, found 396.9619 (**Supplementary Figure 34**).

Preparationof Various Substitutedmethyl N-(tert-butoxycarbonyl)-1-tosyl-L-tryptophanate (4)

Methyl (S)-2-((tert-butoxycarbonyl)amino)-3-(6-cyano-1-tosyl-1*H*-indol-3-yl)propanoate (**4a**)

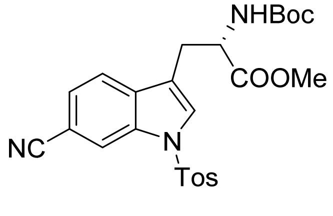

4a

Methyl (S)-2-((tert-butoxycarbonyl)amino)-3-(7-cyano-1-tosyl-1*H*-indol-3-yl)propanoate (**4b**)

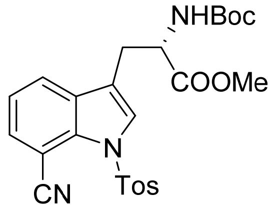

4b

1,2-Dibromoethane (282 mg, 1.5 mmol) was added to a stirred suspension of zinc dust (3.25 g, 50mmol) in DMF (40 mL), and the mixture was stirred at 50 °C for 30 min. The reaction mixture was allowed to cool to room temperature. Chlorotrimethylsilane (54 mg, 0.5 mmol) was added to the mixture, and the mixture was stirred for a further 30 min vigorously. Boc-3-iodo-L-alanine methyl ester (4.11 g, 12.5 mmol) in DMF (20 mL) was added to the reaction mixture, which was then stirred at room temperature for 2 h. The supernatant liquid was transferred to the compound **3** (1.88 g, 5 mmol), $Pd(OAc)_2$ (112 mg, 0.5 mmol), and S-Phos (205 mg, 0.5 mmol) via syringe. The reaction mixture was stirred at 35°C for 4 h at Argon atmosphere. After pouring into water, the mixture was extracted with EtOAc. The combined organic layer was washed with brine, dried over Na_2SO_4, and concentrated. The residue was purified by column chromatography using PE and EtOAc to afford the compound **4a** and **4b**. **4a**) White solid (1.02 g, 41%). ^{1}H NMR (500 MHz, DMSO-d_6) δ 8.32 (s, 1H), 7.97 – 7.91 (m, 3H), 7.80 (d, J = 8.2 Hz, 1H), 7.68 (d, J = 12.4 Hz, 1H), 7.39 (m, 3H), 4.30 (td, J = 9.8, 5.0 Hz, 1H), 3.58 (s, 3H), 3.15 (dd, J = 14.7, 4.8 Hz, 1H), 3.01 (dd, J = 14.7, 10.1 Hz, 1H), 2.33 (s, 3H), 1.27 (s, 9H) (**Supplementary Figure 35**). ^{13}C NMR (126 MHz, DMSO-d_6) δ 172.55 (s), 155.73 (s), 146.38 (s), 134.34 (s), 134.22 (s), 133.66 (s), 130.88 (s), 128.89 (s), 127.43 (s), 126.91 (s), 121.68 (s), 119.75 (s), 119.14 (s), 117.61 (s), 107.13 (s), 78.83 (s), 60.23 (s), 53.76 (s), 52.38 (s), 28.44 (s), 21.51 (s) (**Supplementary Figure 36**). HRMS (ESI) m/z calcd. for $C_{25}H_{27}N_3NaO_6S^+$ $(M+Na)^+$ 520.1513, found 520.1513 (**Supplementary Figure 37**).

4b) White solid (1.19 g, 48%). ^{1}H NMR (500 MHz, DMSO-d_6) δ 8.00 (d, J = 7.9 Hz, 1H), 7.91 (s, 1H), 7.83 (d, J = 8.3 Hz, 2H), 7.78 (d, J = 7.4 Hz, 1H), 7.42 (d, J = 8.1 Hz, 4H), 4.41 – 4.31 (m, 1H), 3.63 (s, 3H), 3.20 (dd, J = 14.8, 4.7 Hz, 1H), 3.07 (dd, J = 14.8, 10.2 Hz, 1H), 2.34 (s, 3H), 1.28 (s, 9H) (**Supplementary Figure 38**). ^{13}C NMR (126 MHz, DMSO-d_6) δ 172.58 (s), 155.78 (s), 146.23 (s), 134.74 (s), 133.28 (s), 132.87 (s), 131.42 (s), 130.63 (s), 128.41 (s), 127.62 (s), 126.07 (s), 124.06 (s), 118.29 (s), 117.28 (s), 97.57 (s), 78.85 (s), 53.74 (s), 52.42 (s), 28.44 (s), 26.16 (s), 21.51 (s) (**Supplementary Figure 39**). HRMS (ESI) m/z calcd. for $C_{25}H_{27}N_3NaO_6S^+$ $(M+Na)^+$ 520.1513, found 520.1516 (**Supplementary Figure 40**).

Preparation of Various Substituted L-tryptophan hydrochloride (5)

(S)-2-amino-3-(6-cyano-1*H*-indol-3-yl)propanoic acid hydrochloride (**5a**)

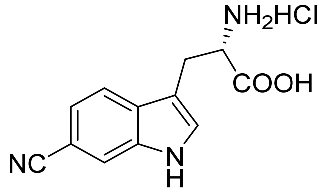

5a

(S)-2-amino-3-(7-cyano-1*H*-indol-3-yl)propanoic acid hydrochloride (**5b**)

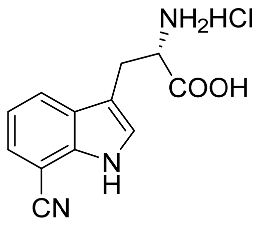

5b

The compound **4a** and **4b** (994 mg, 2 mmol) were dissolved in EtOH (30 mL) respectively, and NaOH (240 mg, 6 mmol) in H_2O (10 mL) was added. The mixture was heated under reflux for 3 h and then the EtOH was evaporated under reduced pressure. The acidic product was recovered by extraction of the water phase with ethyl acetate to pH 3 and the

solvent was removed. The solid was dissolved in 1,4-dioxane (20 mL), and hydrochloric acid (6 mL) was added. The mixture was stirred at room temperature for 4 h. The solvent was evaporated under reduced pressure and the compound **5a** or **5b** was obtained by recrystallization with diethyl ether and EtOH. **5a**) White solid (318 mg, 60 %). ^{1}H NMR (500 MHz, DMSO-d_6) δ 11.73 (s, 1H), 8.38 (s, 3H), 7.89 (d, J = 0.7 Hz, 1H), 7.77 (d, J = 8.3 Hz, 1H), 7.57 (d, J = 2.5 Hz, 1H), 7.36 (dd, J = 8.3, 1.4 Hz, 1H), 4.16 (t, J = 5.6 Hz, 1H), 3.33 (d, J = 6.0 Hz, 2H) (**Supplementary Figure 41**). ^{13}C NMR (126 MHz, DMSO-d_6) δ 171.17 (s), 135.47 (s), 130.70 (s), 130.20 (s), 121.75 (s), 121.08 (s), 120.03 (s), 116.90 (s), 108.41 (s), 102.92 (s), 52.99 (s), 26.04 (s) (**Supplementary Figure 42**). HRMS (ESI) m/z calcd. for $C_{12}H_{10}N_3O_2^-$ (M-H)$^-$ 228.0779, found 228.0784 (**Supplementary Figure 43**). **5b**) White solid (382 mg, 72 %). 1H NMR (500 MHz, DMSO-d6) δ 12.00 (s, 1H), 8.46 (s, 3H), 7.99 (d, J = 8.0 Hz, 1H), 7.61 (d, J = 6.9 Hz, 1H), 7.44 (d, J = 2.4 Hz, 1H), 7.18 (t, J = 7.7 Hz, 1H), 4.16 (t, J = 5.8 Hz, 1H), 3.36 (d, J = 6.1 Hz, 2H) (**Supplementary Figure 44**). 13C NMR (126 MHz, DMSO-d6) δ 171.13 (s), 136.26 (s), 128.70 (s), 127.82 (s), 126.90 (s), 124.81 (s), 119.33 (s), 117.94 (s), 109.03 (s), 94.09 (s), 52.87 (s), 25.93 (s) (**Supplementary Figure 45**). HRMS (ESI) m/z calcd. for $C_{12}H_{10}N_3O_2^-$ (M-H)$^-$ 228.0779, found 228.0788 (**Supplementary Figure 46**).

Supplementary Figure 24. ^{1}H NMR of compound 2a

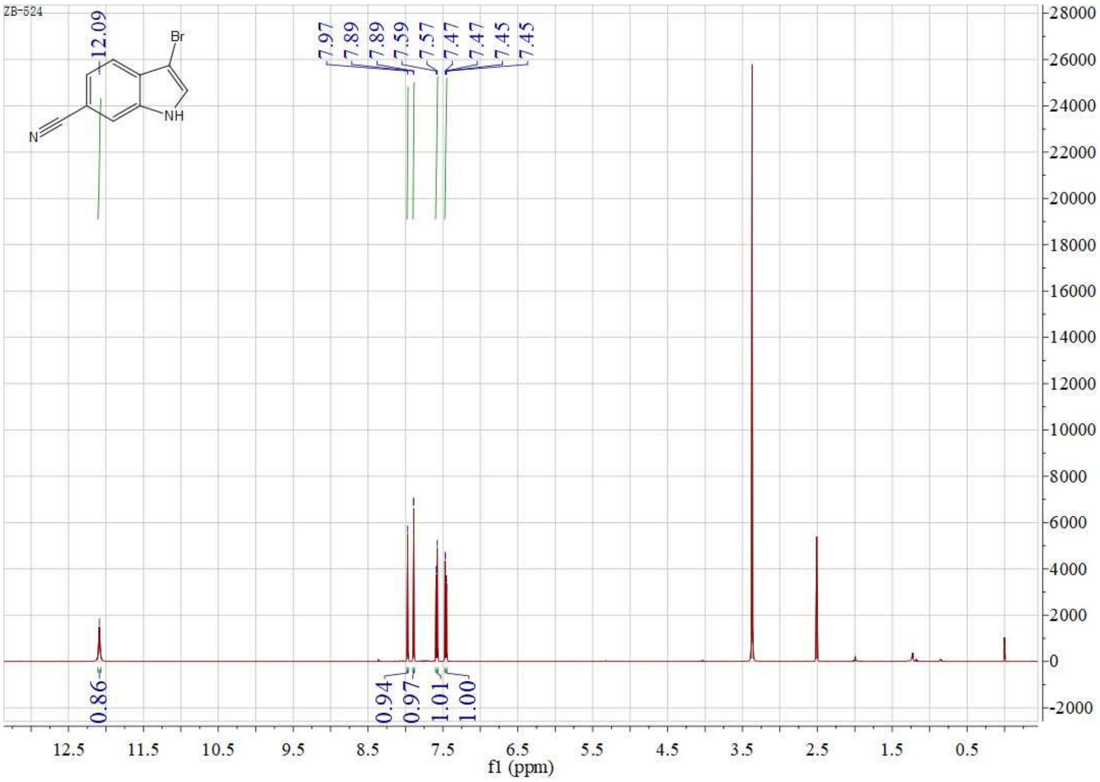

Supplementary Figure 25. HRMS of compound 2a

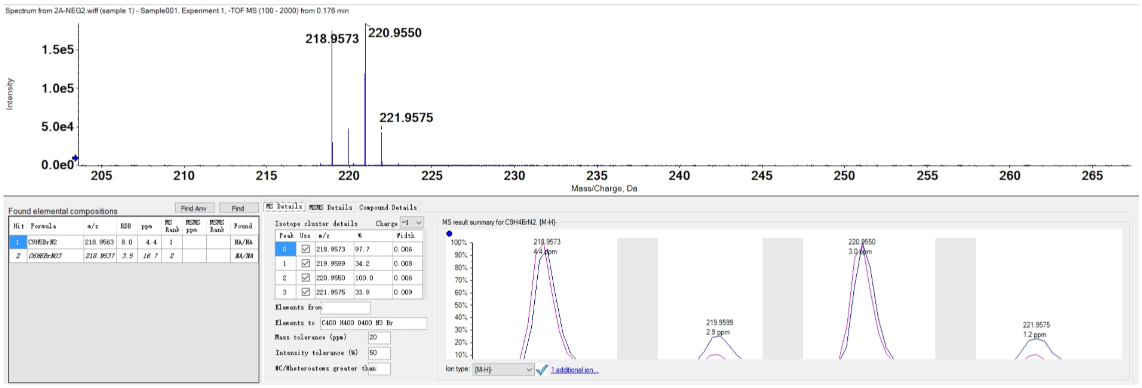

Supplementary Figure 26. ^{1}H NMR of compound 2b

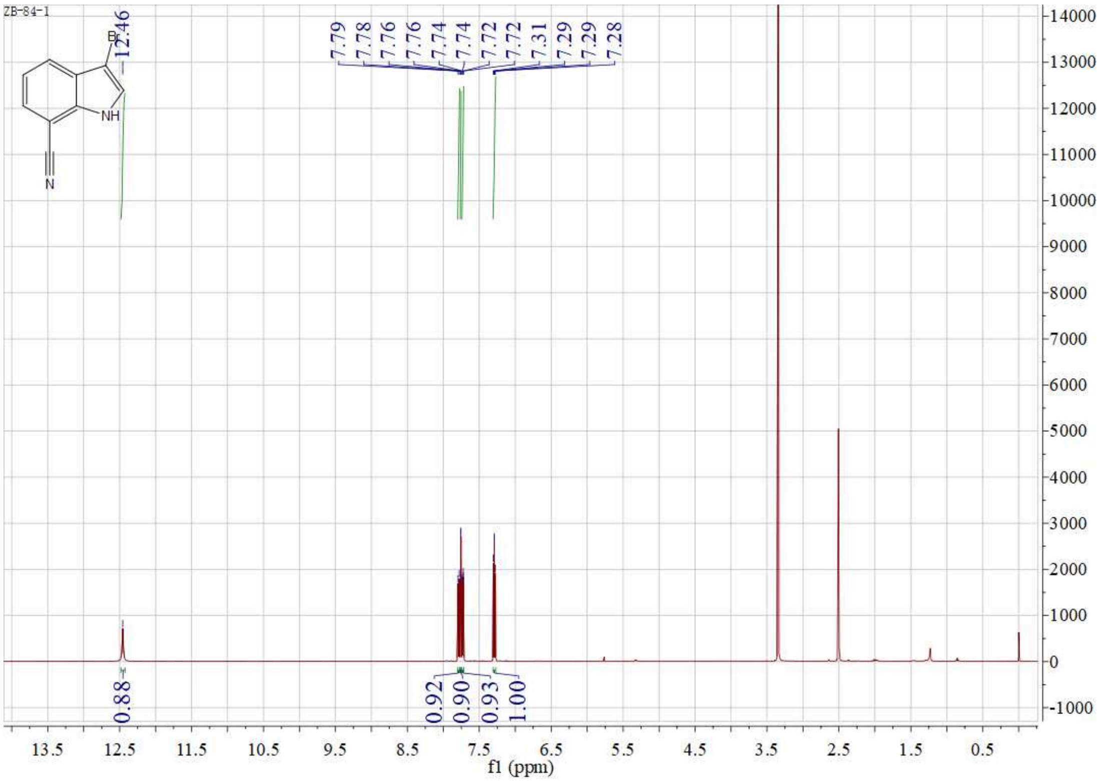

Supplementary Figure 27. ^{13}C NMR of compound 2b

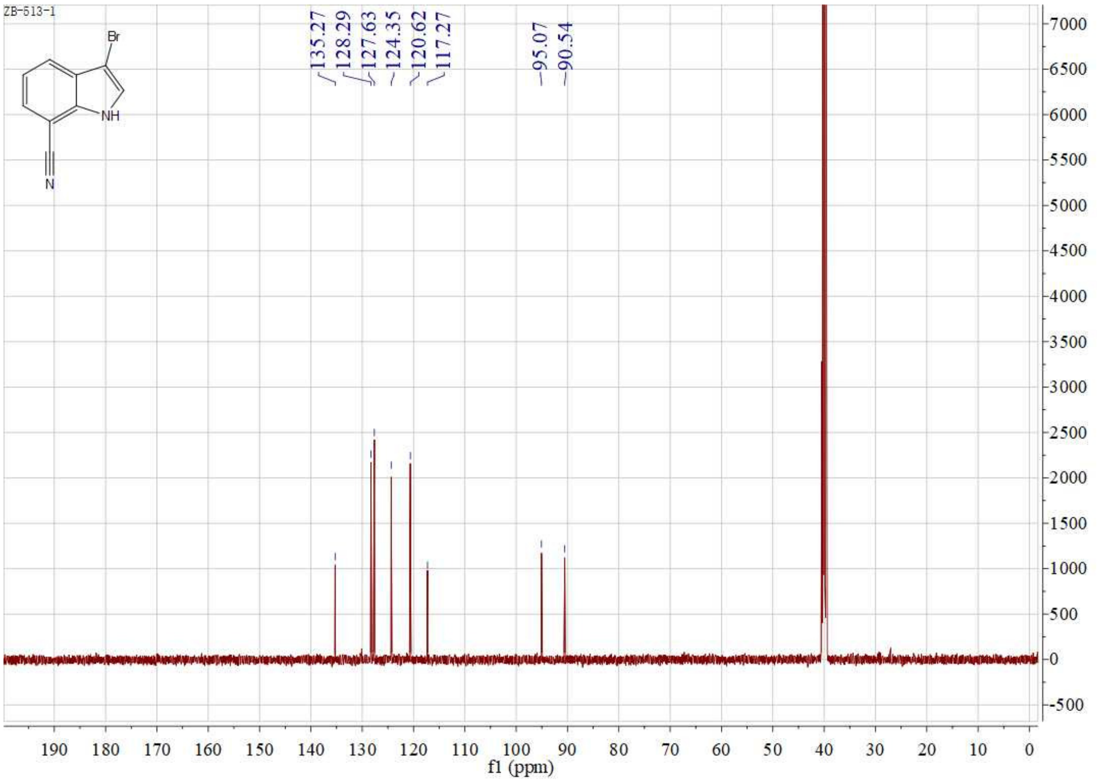

Supplementary Figure 28. HRMS of compound 2b

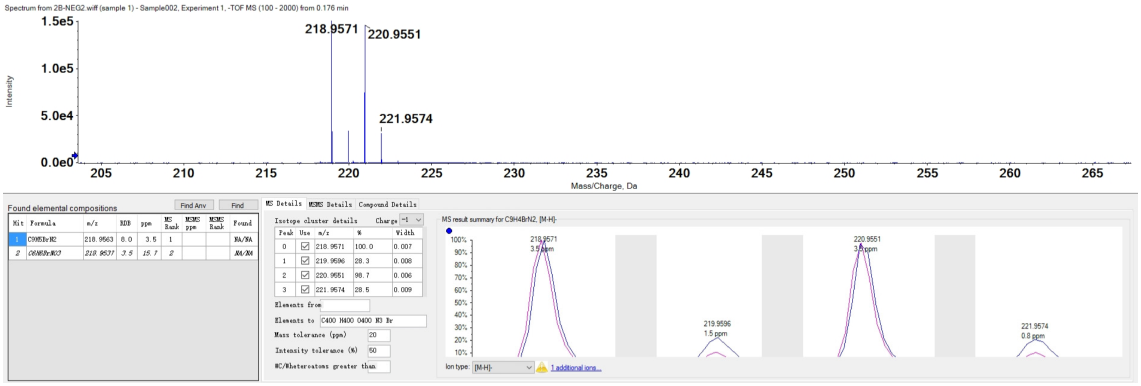

Supplementary Figure 29. ^{1}H NMR of compound 3a

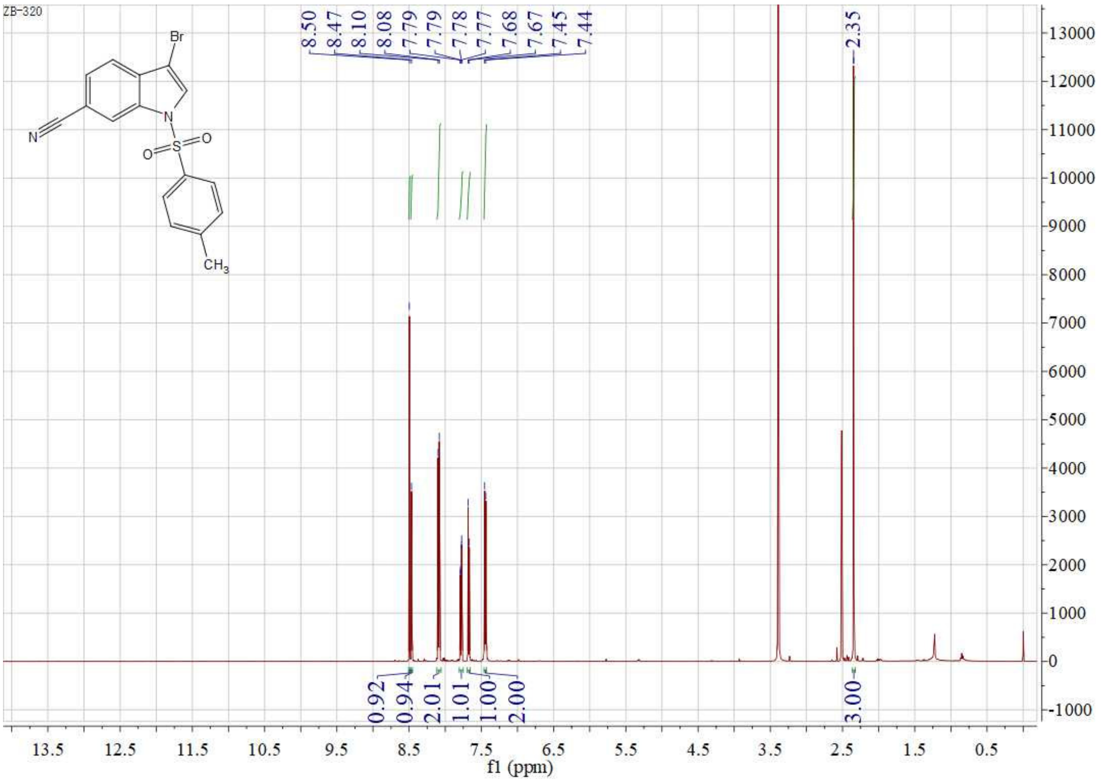

Supplementary Figure 30. ^{13}C NMR of compound 3a

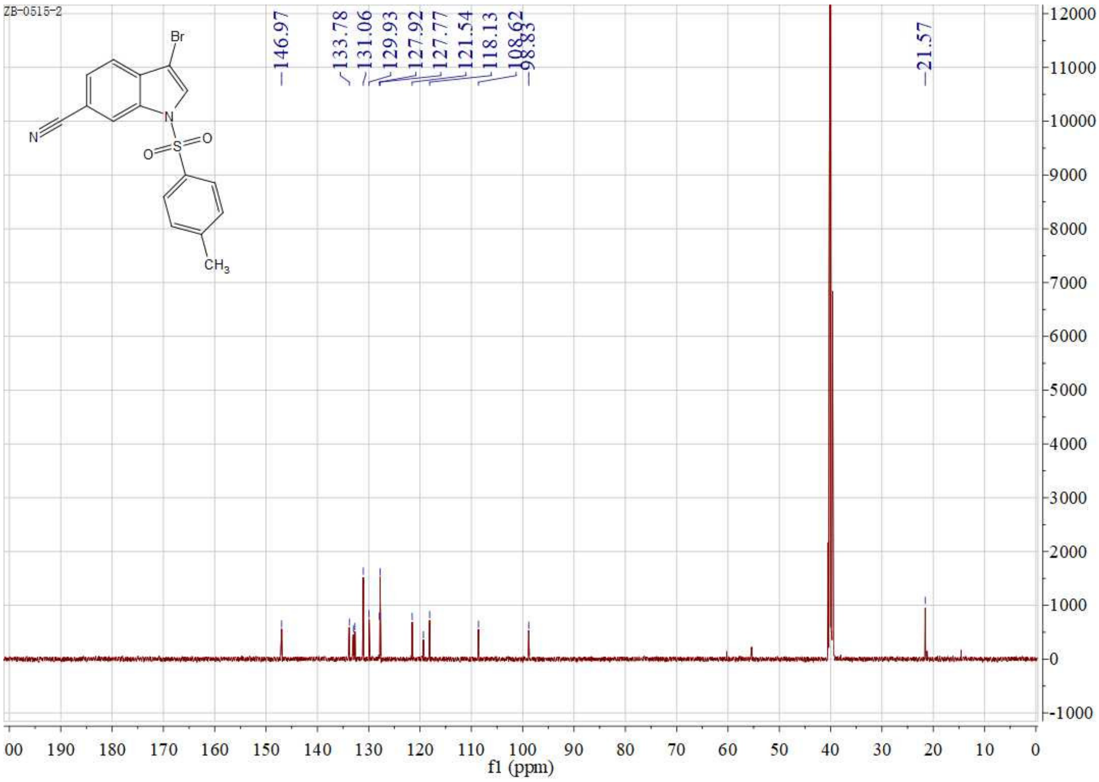

Supplementary Figure 31. HRMS of compound 3a

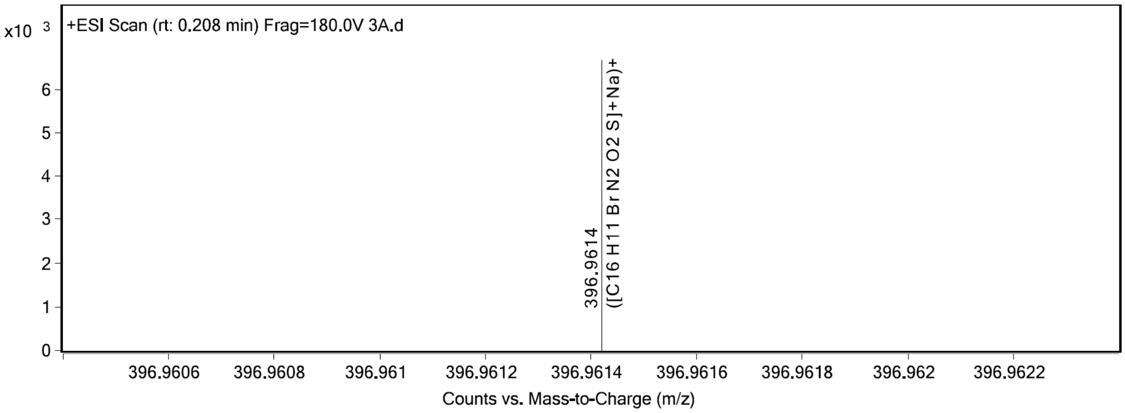

Supplementary Figure 32. ^{1}H NMR of compound 3b

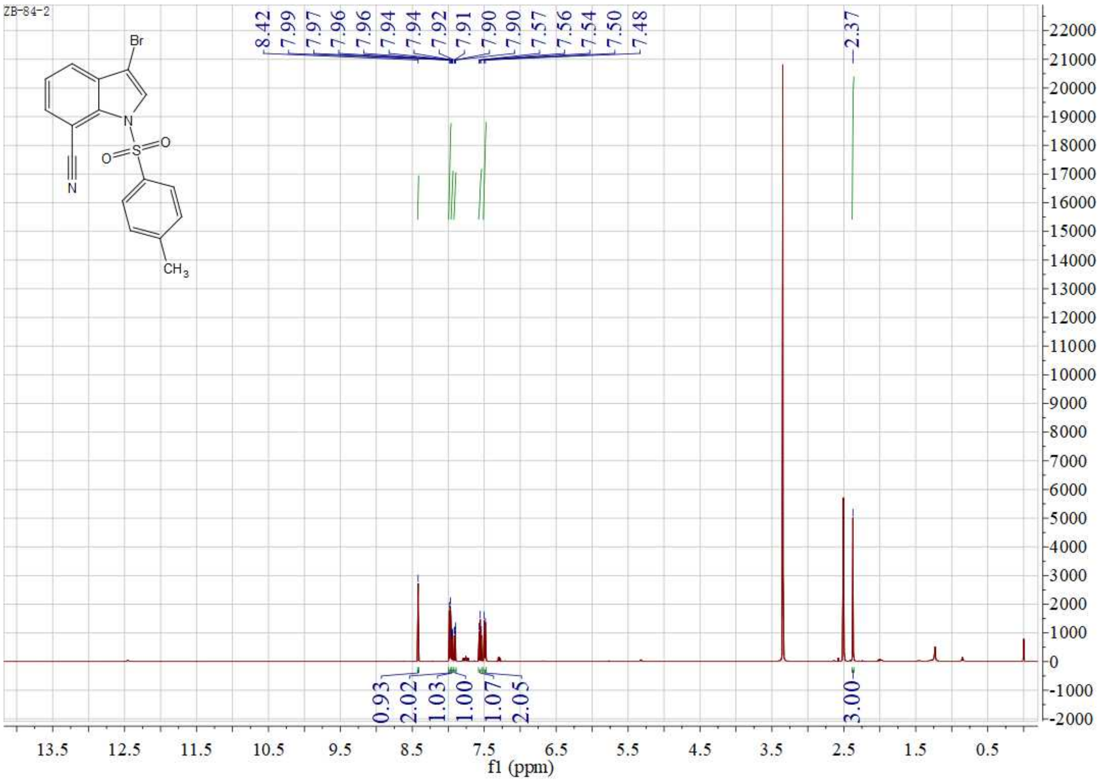

Supplementary Figure 33. ^{13}C NMR of compound 3b

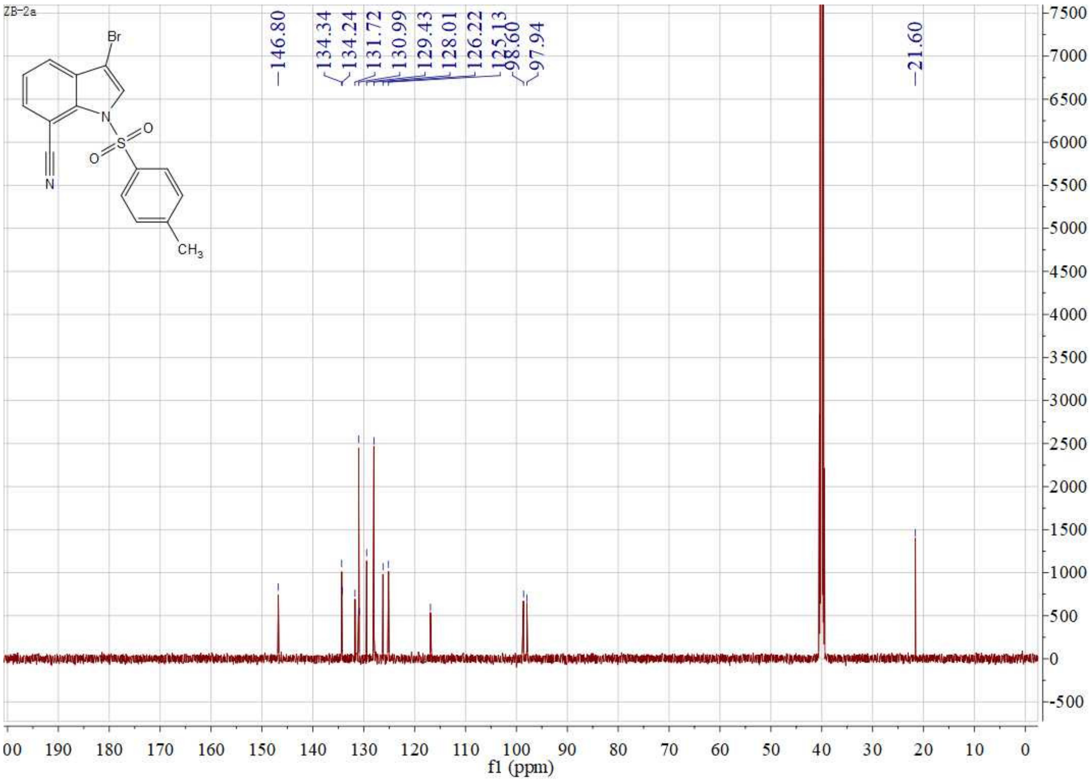

Supplementary Figure 34. HRMS of compound 3b

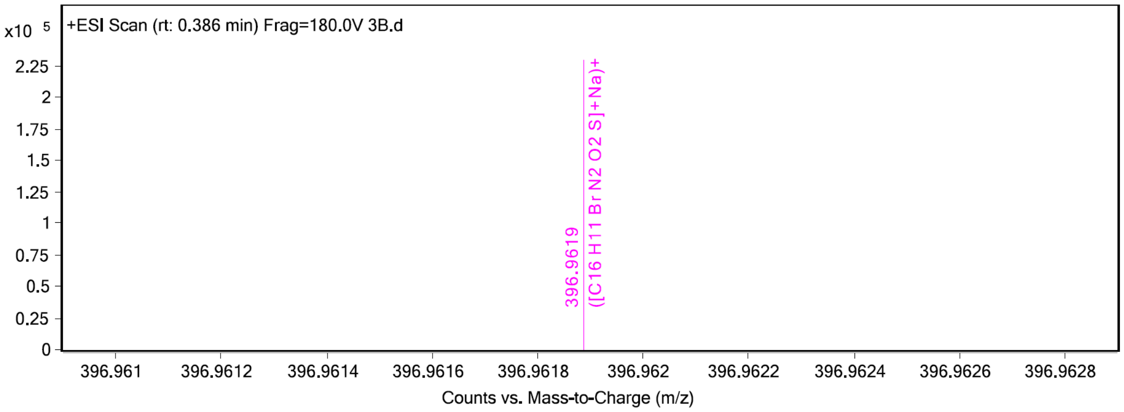

Supplementary Figure 35. ^{1}H NMR of compound 4a

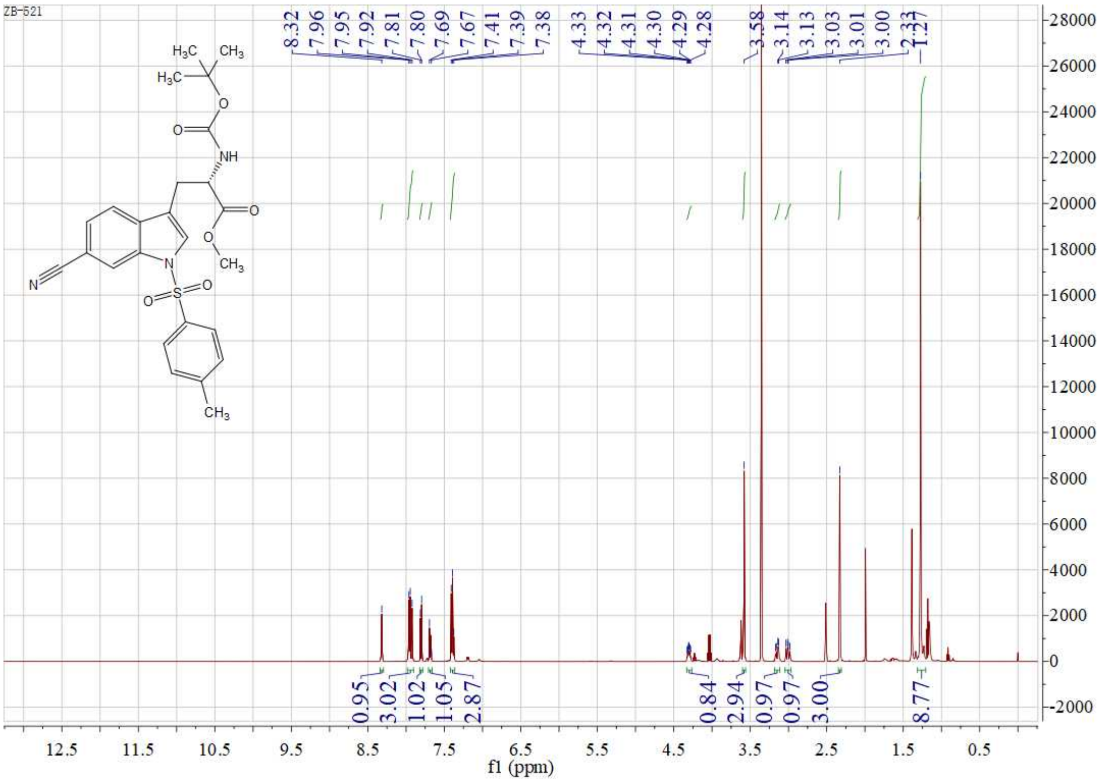

Supplementary Figure 36. ^{13}C NMR of compound 4a

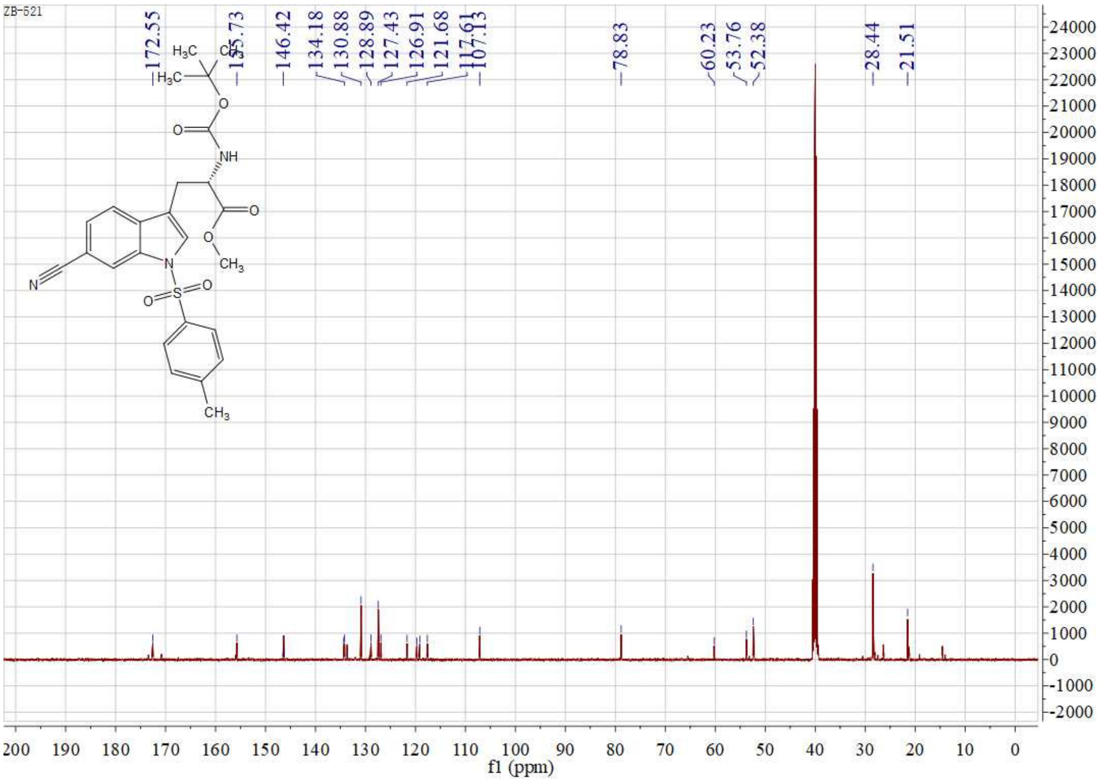

Supplementary Figure 37. HRMS of compound 4a

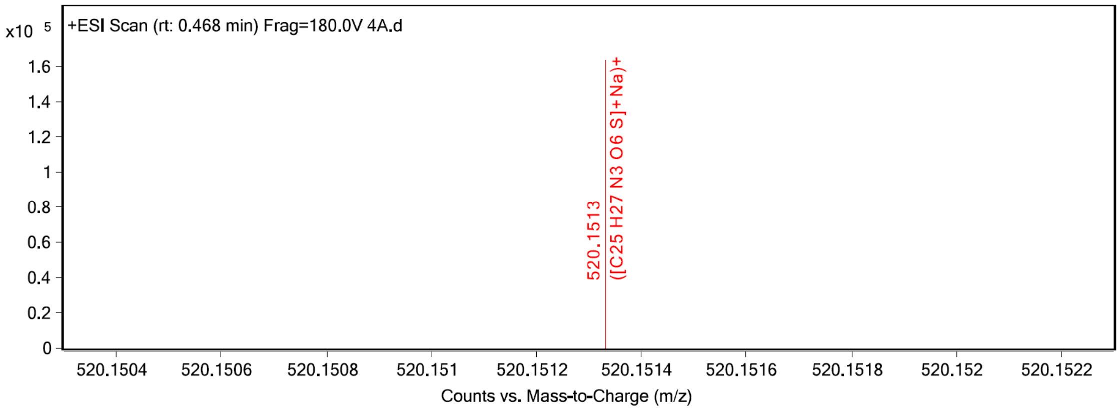

Supplementary Figure 38. ^{1}H NMR of compound 4b

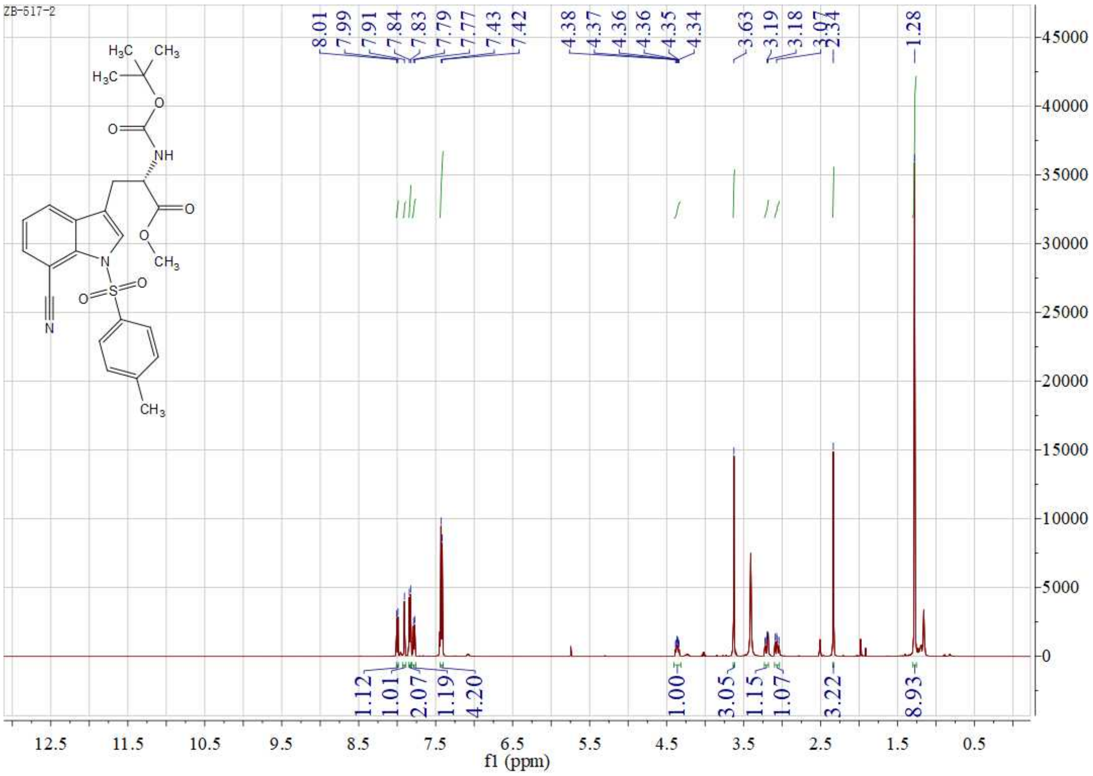

Supplementary Figure 39. ^{13}C NMR of compound 4b

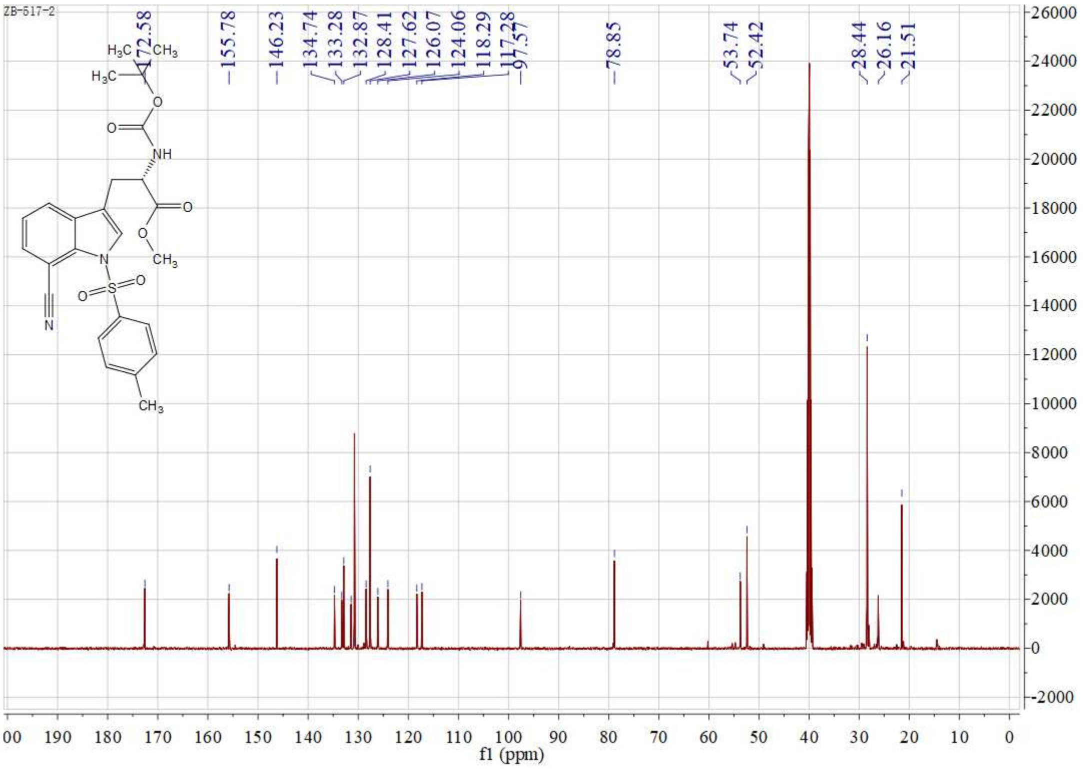

Supplementary Figure 40. HRMS of compound 4b

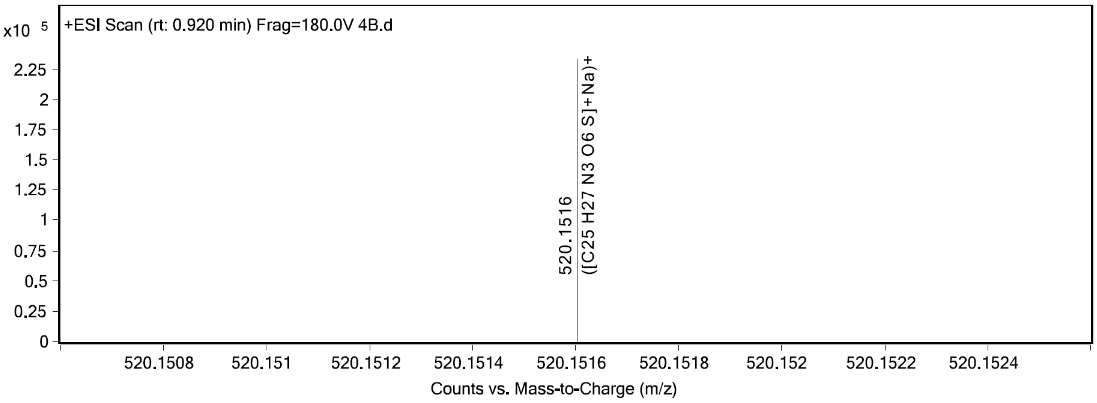

Supplementary Figure 41. ^{1}H NMR of compound 5a

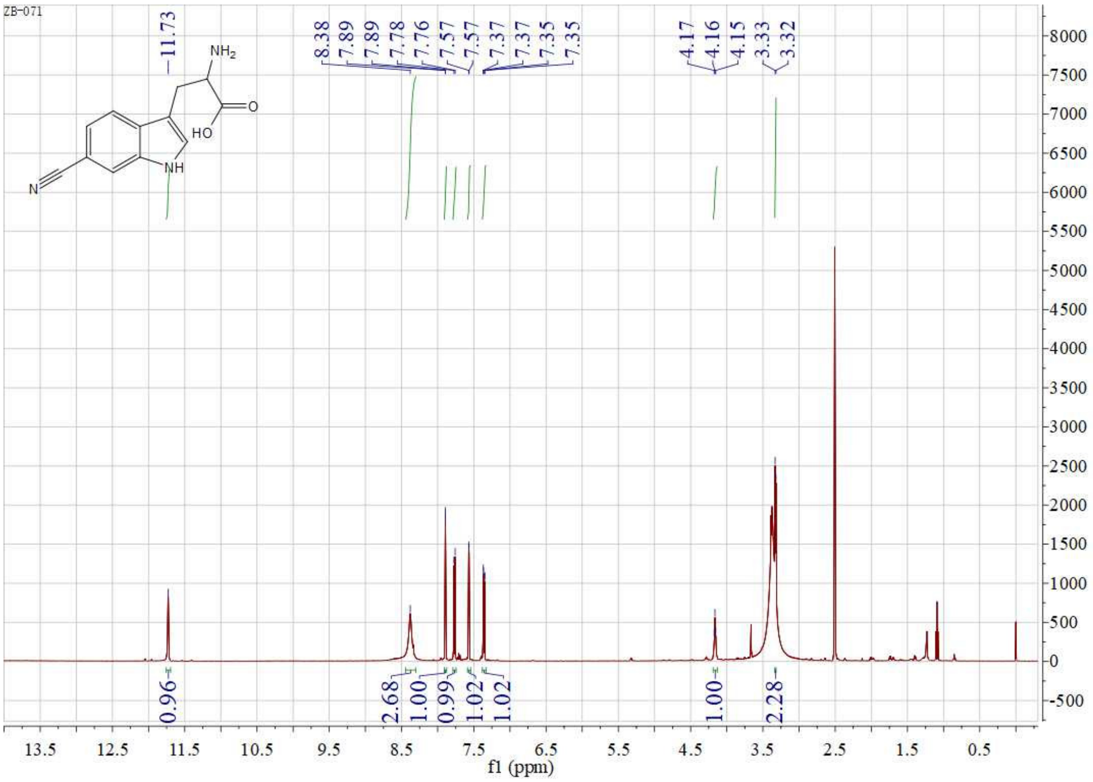

Supplementary Figure 42. ^{13}C NMR of compound 5a

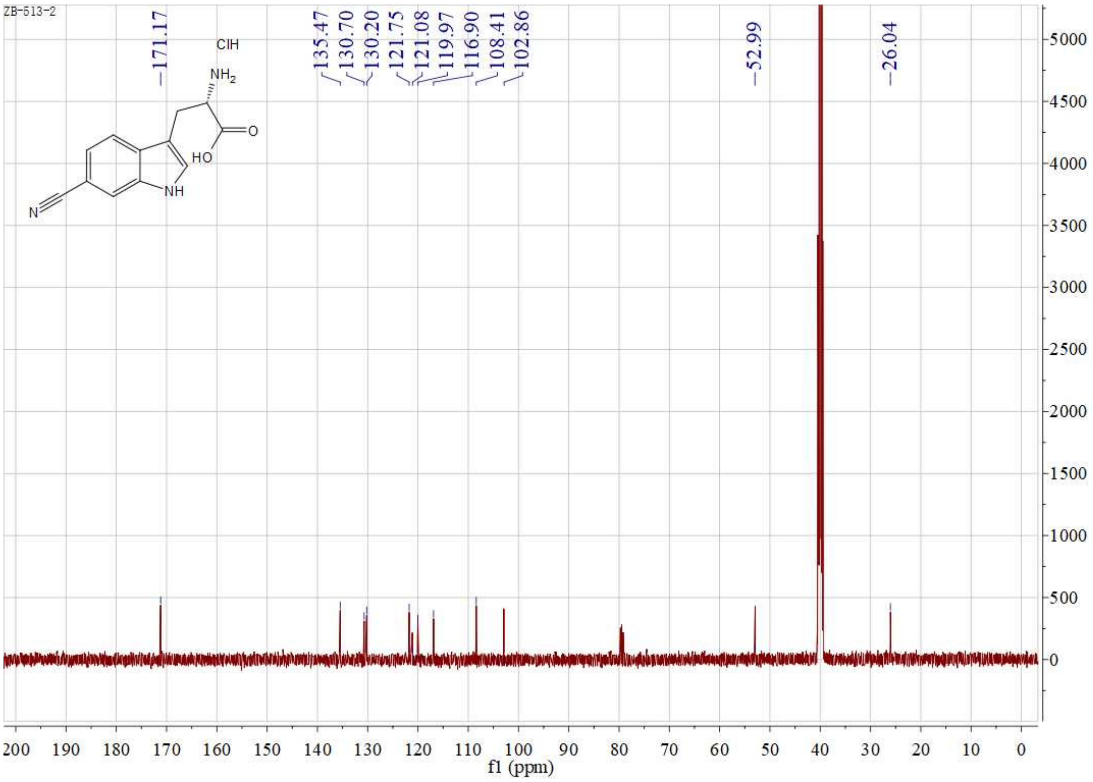

Supplementary Figure 43. HRMS of compound 5a

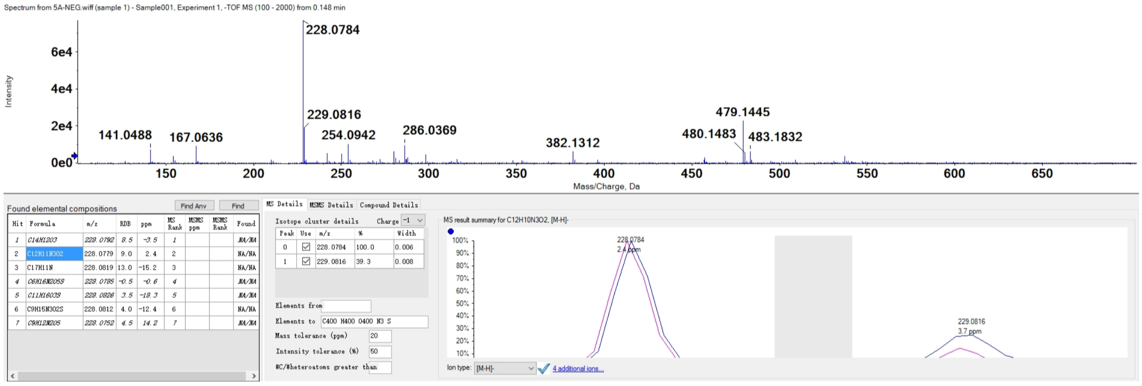

Supplementary Figure 44. ^{1}H NMR of compound 5b

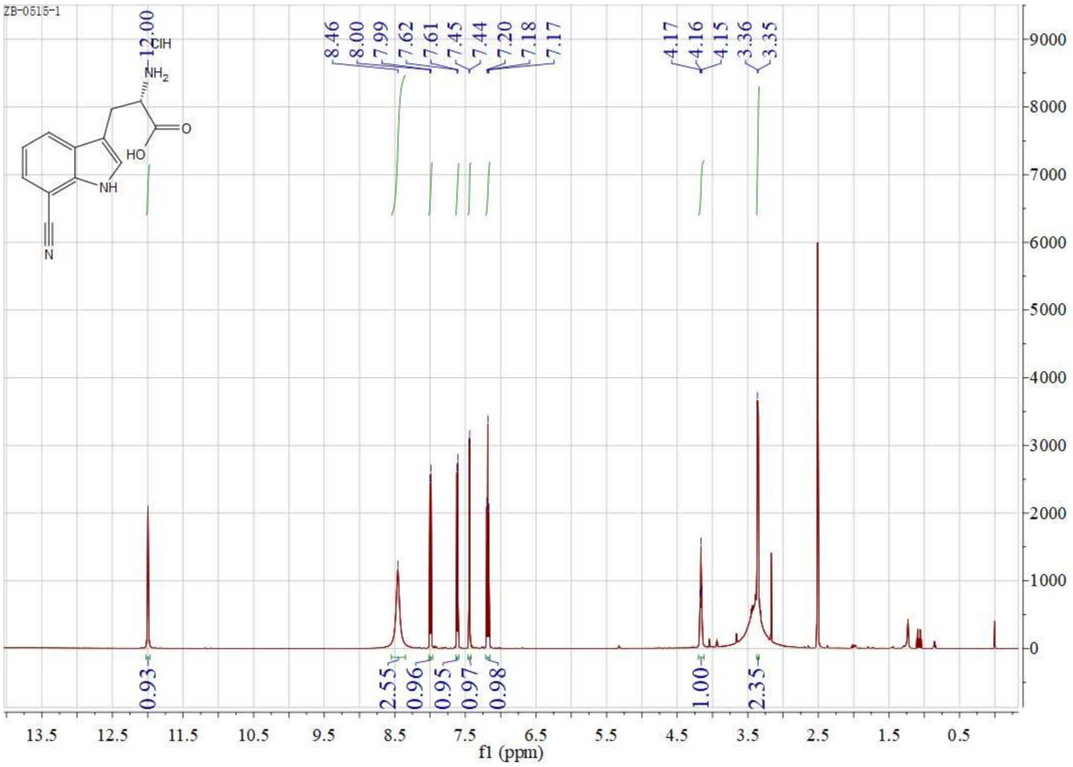

Supplementary Figure 45. ^{13}C NMR of compound 5b

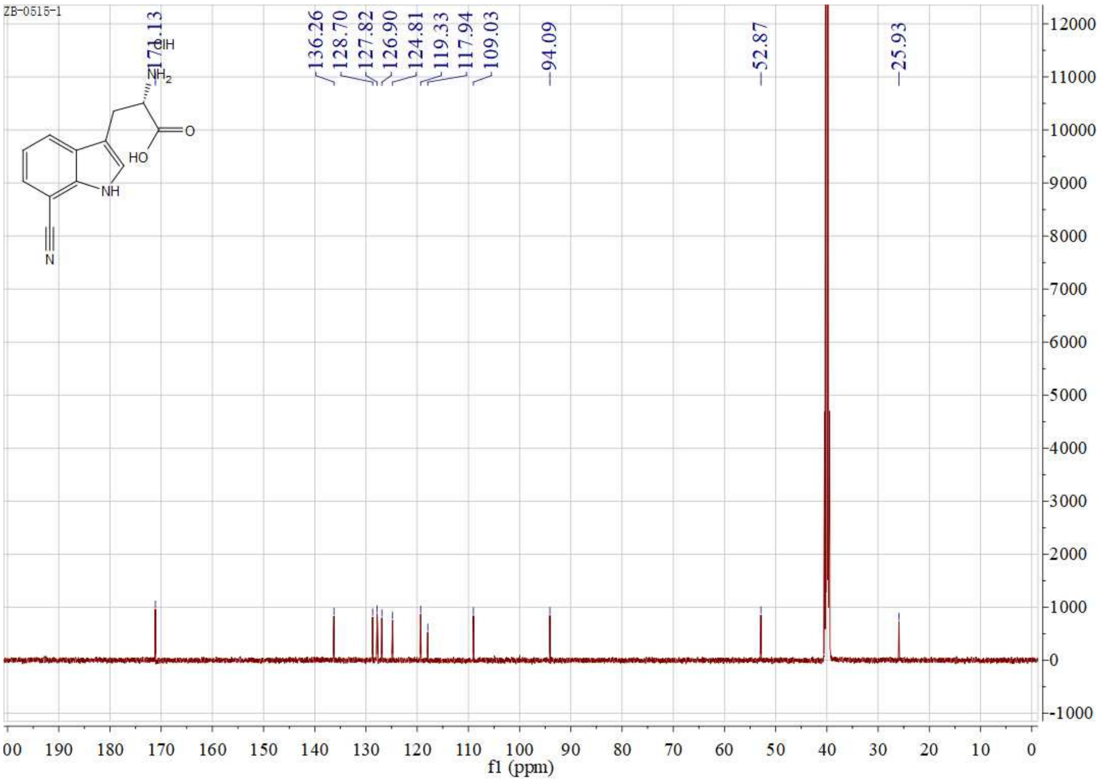

Supplementary Figure 46. HRMS of compound 5b

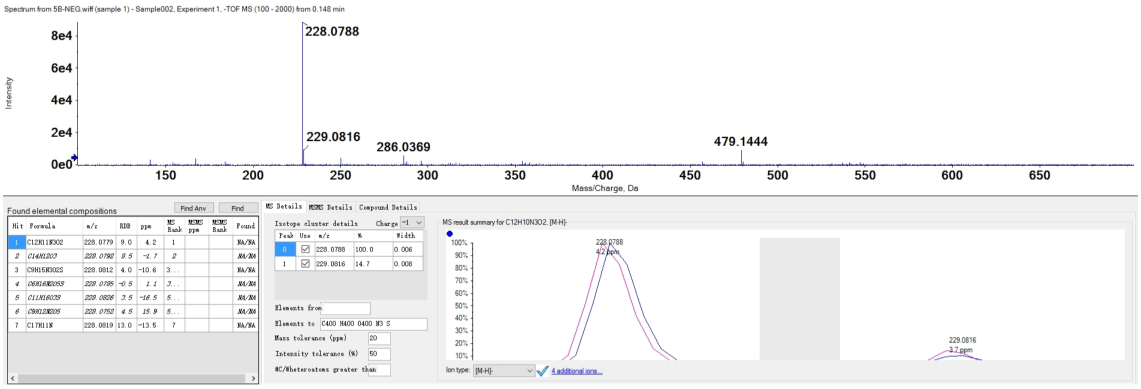

References

1. Cox J, Mann M. MaxQuant enables high peptide identification rates, individualized p.p.b.-range mass accuracies and proteome-wide protein quantification. *Nat. Biotechnol.* 2008, **26**, 1367-1372 (2008).

2. Cox J, Neuhauser N, Michalski A, Scheltema RA, Olsen JV, Mann M. Andromeda: A peptide search engine integrated into the MaxQuant environment. *J. Proteome Res.* **10**, 1794-1805 (2011).

3. Wang LH, Li DQ, Fu Y, Wang HP, Zhang JF, Yuan ZF*, et al.* pFind 2.0: a software package for peptide and protein identification via tandem mass spectrometry. *Rapid Commun. Mass Spectrom.* **21**, 2985-2991 (2007).

4. Perez-Riverol, Y. et al. The PRIDE database and related tools and resources in 2019: improving support for quantification data. *Nucleic Acids Res.* **47**, D442-D450 (2019).
