# Supplementary material for: Chimeric design of pyrrolysyl-tRNA synthetase/tRNA pairs and canonical synthetase/tRNA pairs for genetic code expansion
Source: Nat Commun. 2020 Jun 22;11:3154. doi: 10.1038/s41467-020-16898-y (PMC7308279; doi:10.1038/s41467-020-16898-y)
Supplement: Supplementary file 2 — Reporting Summary [file 41467_2020_16898_MOESM2_ESM.pdf]

nature research

Corresponding author(s): Shixian Lin

Last updated by author(s): May 26, 2020

Reporting Summary

Nature Research wishes to improve the reproducibility of the work that we publish. This form provides structure for consistency and transparency in reporting. For further information on Nature Research policies, see our Editorial Policies and the Editorial Policy Checklist.

Statistics

For all statistical analyses, confirm that the following items are present in the figure legend, table legend, main text, or Methods section.

n/a	Confirmed	
☐	☒	The exact sample size (*n*) for each experimental group/condition, given as a discrete number and unit of measurement
☐	☒	A statement on whether measurements were taken from distinct samples or whether the same sample was measured repeatedly
☐	☒	The statistical test(s) used AND whether they are one- or two-sided<br>*Only common tests should be described solely by name; describe more complex techniques in the Methods section.*
☒	☐	A description of all covariates tested
☒	☐	A description of any assumptions or corrections, such as tests of normality and adjustment for multiple comparisons
☐	☒	A full description of the statistical parameters including central tendency (e.g. means) or other basic estimates (e.g. regression coefficient) AND variation (e.g. standard deviation) or associated estimates of uncertainty (e.g. confidence intervals)
☐	☒	For null hypothesis testing, the test statistic (e.g. *F*, *t*, *r*) with confidence intervals, effect sizes, degrees of freedom and *P* value noted<br>*Give P values as exact values whenever suitable.*
☒	☐	For Bayesian analysis, information on the choice of priors and Markov chain Monte Carlo settings
☒	☐	For hierarchical and complex designs, identification of the appropriate level for tests and full reporting of outcomes
☒	☐	Estimates of effect sizes (e.g. Cohen's *d*, Pearson's *r*), indicating how they were calculated

Our web collection on statistics for biologists contains articles on many of the points above.

Software and code

Policy information about availability of computer code

Data collection	For the assessment of amber suppression efficiency, GFP fluorescence were collected with Gen5 CHS 2.09 software. The two-photon imaging were captured by Olympus FV31S-SW. FACS of HEK 293T cells were acquired with CytExpert. Protein gels, including Coomassie blue staining gels and fluorescent gels, and western blotting PVDF membranes were captured by cSeries Capture Software.
Data analysis	The assessment of activity for chimeric pairs were processed with Origin 8.0 software. Comparisons of activity for UAA incorporation with different orthogonal pairs were performed using one-way ANOVA with significance level at α = 0.05 in Origin 8.0 software. All images were analysed and processed with ImageJ software (National Institutes of Health). Mass spectral deconvolution was performed using UNIFI software (version 1.9.4, Waters Corporation).The MS/MS raw files were processed with MaxQuant software (version 1.6.14.0). FACS data were processed with FlowJo (Treestar Software).

For manuscripts utilizing custom algorithms or software that are central to the research but not yet described in published literature, software must be made available to editors and reviewers. We strongly encourage code deposition in a community repository (e.g. GitHub). See the Nature Research guidelines for submitting code & software for further information.

Data

Policy information about availability of data

All manuscripts must include a data availability statement. This statement should provide the following information, where applicable:

- Accession codes, unique identifiers, or web links for publicly available datasets
- A list of figures that have associated raw data
- A description of any restrictions on data availability

Any Supplementary Information (methods and figures), Supplementary Data (DNA sequences and protein sequences), and chemical compound information are available in the online version of the paper. LC-MS/MS data underlying Supplementary Figures 3C, 13E, 15G, 16D, 19D, and 22C-D have been deposited to the

ProteomeXchange Consortium via the PRIDE partner repository with the dataset identifier PXD018660 [https://www.ebi.ac.uk/pride/archive/projects/PXD018660]. The source data underlying Figure 2D, 3B-E, 4A-G, 5C-F, 6B-F, and Supplementary Figures 6B, 7, 10B, 13A, 14, 15B-C, 16B-C, 17C-E, 19B-C, 20A-G, 21 are provided as a Source Data file. All relevant data are available from the corresponding author upon reasonable request. Correspondence and requests for materials should be addressed to S.L.

Field-specific reporting

Please select the one below that is the best fit for your research. If you are not sure, read the appropriate sections before making your selection.

- [x] Life sciences
- [ ] Behavioural & social sciences
- [ ] Ecological, evolutionary & environmental sciences

For a reference copy of the document with all sections, see nature.com/documents/nr-reporting-summary-flat.pdf

Life sciences study design

All studies must disclose on these points even when the disclosure is negative.

Sample size	Sample size (n=x biologically independent experiments) for each experiment is indicated in the figure legend. In vitro aminoacyltion assays were performed in duplicate due to the use of radioactivity compound. Quantitative fluorescence measurement were performed at least three times. Statistics was performed for experiment with sample size n>2 and error bar represented ±standard error of the mean. Statistical significance is quantified with ordinary one-way ANOVA. The actual p value is indicated in the figure or legend. All data points was indicated in the dot plots and box plots.
Data exclusions	No data were excluded in this study.
Replication	All experiment was repeated independently at least twice with similar results. At least two different biological replicates were performed for all experiment. Statistics was performed for experiment with sample size n>2 and error bar represented ±standard error of the mean. Statistical significance is quantified with ordinary one-way ANOVA. The actual p value is indicated in the figure or legend. All data points is indicated in the dot plots and box plots.
Randomization	E. coli and mammalian cells used in this study were randomly assigned as controls or treatment groups.
Blinding	The investigators collecting and analyzing data like imaging, fluorescent intensity data in this study were blind to the experimental conditions. For other experiment, the investigators were not blinded to sample identity, because the samples form defined groups.

Reporting for specific materials, systems and methods

We require information from authors about some types of materials, experimental systems and methods used in many studies. Here, indicate whether each material, system or method listed is relevant to your study. If you are not sure if a list item applies to your research, read the appropriate section before selecting a response.

Materials & experimental systems

n/a	Involved in the study	
[ ]	[x]	Antibodies
[ ]	[x]	Eukaryotic cell lines
[x]	[ ]	Palaeontology and archaeology
[x]	[ ]	Animals and other organisms
[x]	[ ]	Human research participants
[x]	[ ]	Clinical data
[x]	[ ]	Dual use research of concern

Methods

n/a	Involved in the study	
[x]	[ ]	ChIP-seq
[ ]	[x]	Flow cytometry
[x]	[ ]	MRI-based neuroimaging

Antibodies

Antibodies used	Primary Antibodies: Anti-His rabbit polyclonal antibody (Cell Signaling Technology, cat#2365, lot#3), Anti-Flag mouse monoclonal antibody (Sigma-Aldrich, cat#F1804, lot# SLCC6485), Anti-GFP rabbit polyclonal antibody (Cell Signaling Technology, cat#2555, lot#6), and Anti-Alpha-Tubulin rat polyclonal antibody (Santa Cruz Biotechnology, cat#sc-53029, lot#C1517) were used in a dilution of 1:1000.<br>Secondary Antibodies: Goat anti-rat IgG (H+L), HRP conjugate (Proteintech, cat#SA00001-15, lot#20000011), Goat anti-mouse IgG (H+L), HRP conjugate (Proteintech, cat#SA00001-1, lot#20000216), and Goat anti-rabbit IgG (H+L), HRP conjugate (Abmart, cat#M21002, lot#303571) were used in a dilution of 1:5000.
Validation	All primary antibodies were validated by the suppliers and used for western blot in this study. Anti-His rabbit antibody was validated by western blot, immunoprecipitation, and by manufacturer provided citations (82 citations). Anti-Flag mouse mAb was validated by western blot and by manufacturer provided citations (4901 citations). Anti-GFP rabbit antibody was validated by western blot, immunohistochemical analysis, and by manufacturer provided citations (112 citations). Anti-Alpha-Tubulin rabbit antibody was validated by western blot, immunohistochemical analysis, immunofluorescence staining, and by manufacturer provided citations (10

citations).

Eukaryotic cell lines

Policy information about cell lines

Cell line source(s)	HEK293T is from ATCC
Authentication	The cell line was used for testing protein expression only. So the cell line was not authenticated.
Mycoplasma contamination	HEK 293T cell line tested negative for mycoplasma contamination.
Commonly misidentified lines (See ICLAC register)	No commonly misidentified cell lines were used in the study.

Flow Cytometry

Plots

Confirm that:

- [x] The axis labels state the marker and fluorochrome used (e.g. CD4-FITC).
- [x] The axis scales are clearly visible. Include numbers along axes only for bottom left plot of group (a 'group' is an analysis of identical markers).
- [x] All plots are contour plots with outliers or pseudocolor plots.
- [x] A numerical value for number of cells or percentage (with statistics) is provided.

Methodology

Sample preparation	For FACS analysis of live cells, HEK 293T cells were co-transfected the pcDNA3.1 vector bearing the aaRS/tRNA pair (pcDNA vector bearing chimeric tRNA alone as a negative control) and pEGFP-mCherry-T2AGFP190TAG-His6.
Instrument	Beckman CytoFlex
Software	FlowJo
Cell population abundance	At least 50000 single cells were analysed per condition. GFP fluorescence was acquired at the FITC channel, and mCherry fluorescence was acquired at the PE channel. Purity of HEK 293T cells was assessed by cytometry directly after sort employing the same gating strategy used for gating. Purity of HEK 293T cells was generally 99% or higher.
Gating strategy	HEK 293T cells were used to set appropriate forward scatter (FSC) and side scatter (SSC) gains. The fluorescent protein expressed cells were used to set FITC and PE gains and gate. The gating strategy was shown in supplementary Figure 8A.

- [x] Tick this box to confirm that a figure exemplifying the gating strategy is provided in the Supplementary Information.
