# Supplementary material for: Chimeric design of pyrrolysyl-tRNA synthetase/tRNA pairs and canonical synthetase/tRNA pairs for genetic code expansion
Source: Nat Commun. 2020 Jun 22;11:3154. doi: 10.1038/s41467-020-16898-y (PMC7308279; doi:10.1038/s41467-020-16898-y)
Supplement: Supplementary file 3 — Description of Additional Supplementary Files [file 41467_2020_16898_MOESM3_ESM.pdf]

Description of Additional Supplementary Files

Title: Supplementary Data 1
Description: DNA sequences and protein sequences used in this study
